# To cut or not to cut? Extended mesenteric excision during intestinal resection does not impact the postoperative recurrence nor the postoperative complications in Crohn’s disease: a systematic review and meta-analysis

**DOI:** 10.1007/s10151-025-03110-w

**Published:** 2025-03-08

**Authors:** M. Topala, P. Martinekova, A. Rancz, D. S. Veres, K. Lenti, P. Miheller, B. Erőss, P. Hegyi, S. Ábrahám

**Affiliations:** 1 Carol Davila University of Medicine and Pharmacy, Bucharest, Romania; 2 Centre For Translational Medicine, Semmelweis University, Üllői út 26, Budapest, 1085 Hungary; 3 EDU Institute of Higher Education, Medicine and Health, Kalkara, Malta; 4 Department of Internal Medicine and Hematology, Medical School, Semmelweis University, Budapest, Hungary; 5 Department of Biophysics and Radiation Biology, Semmelweis University, Budapest, Hungary; 6 Department of Morphology and Physiology, Faculty of Health Sciences, Semmelweis University, Budapest, Hungary; 7 Department of Surgery, Transplantation and Gastroenterology, Semmelweis University, Budapest, Hungary; 8 Institute for Translational Medicine, Medical School, University of Pécs, Pecs, Hungary; 9 Institute of Pancreatic Diseases, Semmelweis University, Budapest, Hungary; 10 Department of Surgery, Faculty of Medicine, University of Szeged, Szeged, Hungary

**Keywords:** Postoperative recurrence, Creeping fat, Visceral adipose tissue, Inflammatory bowel disease

## Abstract

**Background:**

The mesentery might be involved in the pathogenesis of Crohn’s disease (CD). As a result of scarce and conflicting data, it is debatable whether removal during intestinal resections could influence postsurgical outcome. We aimed to investigate the association between the extent of mesenteric excision during intestinal resections and postoperative complications and recurrence.

**Methods:**

We conducted a systematic search in five databases on 29 July 2024 for studies reporting outcomes in patients with CD who underwent intestinal resections with extended mesenteric excision (EME) compared with limited mesenteric excision (LME). Pooled odds ratios (ORs) with 95% confidence intervals (CI) were calculated using the random-effects model. We assessed the risk of bias using the ROBINS-I and RoB2 tool and evaluated the certainty of evidence according to the GRADE Working Group recommendations.

**Results:**

We retrieved data from six studies, covering 4590 patients. The pooled data showed no significant difference between EME and LME patients regarding surgical recurrence (OR 0.3; 95% CI 0.02–3.73; *p* = 0.176), overall postoperative complications (OR 0.78; 95% CI 0.33–1.82, *p* = 0.329), anastomotic leak (OR 0.76, 95% CI 0.09–6.85, *p* = 0.722), surgical site infection (OR 0.84, 95% CI 0.3–2.36, *p* = 0.539), reoperation rate (OR 1.09, 95% CI 0.33–3.58, *p* = 0.783), or hospitalization (MD − 0.33 (95% CI − 1.8 to 1.15, *p* = 0.53). Individual studies reported similar results regarding 6 months follow-up endoscopic recurrence. The certainty of evidence was very low and low, respectively.

**Conclusion:**

Extended mesenteric excision is not statistically associated with improved postoperative complications or postoperative recurrence. Results should be interpreted cautiously because of the small number of studies; hence, randomized, long-term, controlled trials are needed.

**Supplementary Information:**

The online version contains supplementary material available at 10.1007/s10151-025-03110-w.

## Introduction

Nearly half of patients with Crohn’s disease (CD) are burdened with the development of intestinal complications 20 years after diagnosis [1]. Although therapeutic improvements have reduced surgery rates in recent decades [2], the risk of abdominal surgery after 5 years of illness still varies between 17.3% and 28.9% [3–5].

The main challenge after surgery is the risk of postoperative recurrence (POR). Although endoscopic surveillance and, for patients at higher risk for recurrence, early pharmacological prophylaxis are recommended [6–8], not all patients remain disease-free. Clinical recurrence within 1 year affects 10–38% of patients, whereas endoscopic relapse occurs in 35–85% [9]. Approximately one-fourth of patients require a subsequent resection within 5 years of the initial surgery [10]. Several risk factors for POR have been identified, including patient-related factors (smoking, age ≤ 30 years), disease-related factors (shorter duration, penetrating behavior, perianal disease, extensive intestinal involvement, and prior resections), and histology-related factors (myenteric plexitis, and granulomas) [6–8]. Surgical aspects have also been evaluated as it has been speculated that they might influence postsurgical outcome and certain techniques (e.g., stapled side-to-side and Kono-S anastomoses) have been investigated [11–13]. More recently, the involvement of the mesentery in CD surgery has been explored*.*

The mesentery is associated with bowel alterations in CD and pathological changes, in particular hypertrophy and mesenteric adipose tissue wrapping the intestine in “creeping fat” (CrF), have been described. In terms of its role in modulating the immune-endocrine pathways, it was hypothesized that the mesentery might contribute to the course of CD and, therefore, to postoperative outcome [14]. Thus far, guidelines have not recommended wide mesenterectomy during intestinal resection because of limited evidence [12, 15, 16]. Some studies have reported an association between visceral fat evaluated by cross-sectional imaging and POR [17, 18]; however, data on postoperative complications (POC) have been conflicting [19–21]. Recently, mesenteric excision in Crohn’s surgery has been directly investigated, but studies have reached different conclusions [22–24].

Considering the controversial results reported in the literature and the need to explore whether the mesentery might influence postoperative outcome, both in the short and long term, we performed a systematic review and meta-analysis to evaluate the association between the extent of mesenteric excision during intestinal resections and the risk of POC and POR, respectively.

## Materials and methods

This study was conducted according to the recommendations of the Cochrane Handbook for Systematic Reviews of Interventions Version 6.3, 2022 [25], and reported according to the Preferred Reporting Items for Systematic Reviews and Meta-Analyses (PRISMA) 2020 statement [26] (see Table S1). We followed a pre-established protocol, registered on PROSPERO, registration number CRD42022371789.

### Information sources and search strategy

We searched five databases, MEDLINE (via Pubmed), Scopus, Embase, CENTRAL (The Cochrane Central Register of Controlled Trials), and Web of Science, from inception until 29 July 2024. A TITLE-ABS-KEY filter was used in the Scopus database; however, no other filters or restrictions were applied to other databases.

The search key comprised three domains related to CD and inflammatory bowel diseases, to the mesentery, and to the semantic field of resection and exclusion. The search key for each database can be found in the Supplementary Material.

### Eligibility criteria

We included studies that reported data on postoperative morbidity and POR in patients with CD following intestinal resection with extended mesenteric excision (EME) compared with limited mesenteric excision (LME). Considering the differences between the rectum and the colon regarding the biological behavior and POC, we decided to exclude studies that investigated proctectomy in CD. The clinical question was formulated using the PICO (Population, Intervention, Comparison, Outcomes) framework, where the Population was patients with CD, with the Intervention being intestinal resection with EME (dividing the mesentery close to the origin of the major arterial trunks or the mesenteric root, similar to cancer operation), and the Comparison was bowel resection with LME (dividing the mesentery close to the intestinal wall). We also included surrogate methods that quantified the extent of mesenterectomy, e.g., lymph node yield. The primary outcome was POR, defined as endoscopic, clinical, radiological, and/or surgical recurrence. As secondary outcomes, we evaluated the length of hospital stay and POC (e.g., anastomotic leak, surgical site infections, bleeding, reintervention, general complications, Clavien–Dindo classification). For quantitative analysis, we selected studies that reported the number of events for each outcome or continuous variables for each subgroup. Ethical approval was not required to conduct this study.

### Selection process

All retrieved publications were exported to Endnote™20 reference manager software (Clarivate Analytics, Philadelphia, PA, USA). After automatic and manual duplicate removal, references were uploaded to the Rayyan application [27]. They were screened by title and abstract, and subsequently by full-text content, adhering to eligibility criteria. The references of the selected papers were retrieved on 21 December 2024 using the CitationChaser tool and further surveyed for additional eligible studies [28].

Both the systematic search and the citation search were performed independently by two reviewers (M.T. and P.M.) with any disagreements resolved by a third reviewer (A.R.). The inter-rater reliability was assessed by calculating the Cohen’s kappa coefficient (κ) for each step. Considering the paucity of studies reporting data on the extent of mesenterectomy in CD, we decided to include all relevant published information.

### Data collection process and data items

Two reviewers (M.T. and P.M.) independently collected data in a standardized sheet using Microsoft Excel software (Microsoft Corporation, Redmond, Washington, USA). The extracted information was reported in the Supplementary Material.

### Synthesis methods

Studies included in the quantitative analysis of data could be extracted separately for each intervention and comparator subgroup. A random-effects meta-analysis (as we assumed considerable between-study heterogeneity) was performed for outcomes reported in at least three studies. For binary categorical outcomes, odds ratios (ORs), and for continuous outcomes, mean differences (MDs), with 95% confidence intervals were calculated. See Supplementary Material for complete description of the synthesis methods. All statistical analyses were performed with R software [29] (v4.1.2) using the meta [30] (v6.1.0) package for basic meta-analysis calculations and plots.

### Risk of bias assessment

The Risk Of Bias In Non-randomized Studies of Interventions (ROBINS-I) [31] and the Revised Cochrane Risk Of Bias for Randomized Trials (RoB2) [32] tools were used by two independent reviewers (M.T. and P.M.), and any disagreements were resolved by a third reviewer (A.R.). The domains that were evaluated are detailed in the Supplementary Material. Results were summarized graphically using the Risk-Of-Bias VISualization (robvis) tool [33].

### Certainty of evidence assessment

The assessment was performed according to the risk of bias, inconsistency, indirectness, and imprecision, and according to the recommendations proposed by the Grading of Recommendations, Assessment, Development and Evaluation (GRADE) Working Group [34], using GRADEproGDT software [35].

## Results

### Study selection

**Fig. 1:**
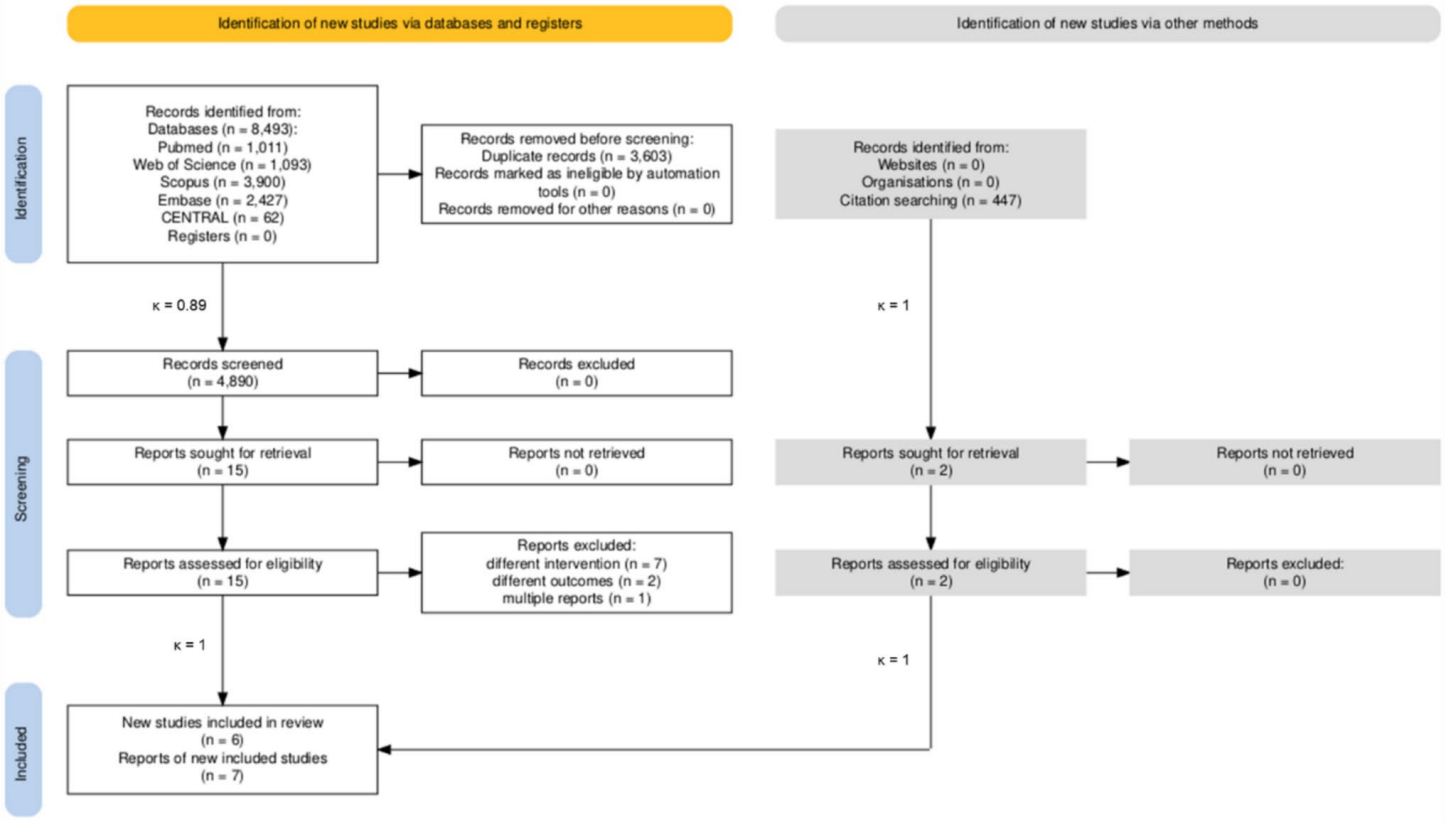
PRISMA 2020 flowchart representing the study selection process

We retrieved 8493 records from databases and 447 from citation search. The study selection process is summarized in the PRISMA 2020 flowchart (Fig. 1).

We included seven papers that reported data from six studies: six articles [22–24, 36–38], and one book chapter [39]. The book chapter was written by Coffey et al., who are also the authors of one of the included journal articles [22] and reported additional data on POC from their original study. The quantitative analysis comprised pooled data from five journal articles [22–24, 37, 38] and from the book chapter [39]. One journal article [36] was included only in the qualitative analysis.

### Study characteristics

The main characteristics of the selected studies are summarized in Table 1. Our study totaled 4590 patients, of whom 4356 were included in the meta-analysis (992 EME, 3364 LME). Patient characteristics are outlined in Table S2 in the Supplementary Material. Baseline characteristics were missing in the studies of Abdulkarim et al. [37] and Ewe et al. [36]. The mean age at the time of the surgery varied between 30 and 42 years old, and the disease duration between 45 months and 7.7 years, with no significant differences between subgroups. There were no significant differences regarding gender, age at diagnosis, or disease location, but dissimilarities in disease behavior were noted in Zhu et al. [23] and Coffey et al. [22] studies. Abdulkarim et al. [37] reported similar rates of comorbidities between subgroups, and only Mineccia et al. [24] reported the presence of extraintestinal manifestations and the indication for surgery. The type of anastomosis was reported by Zhu et al. [23] (stapled side-to-side), Mineccia et al. [24] (28.9% manual and 71.1% stapled end-to-end in EME; 100% manual side-to-side in LME), and de Willebois et al. [38] (side-to-side anastomosis, wide-stapled 99% in EME and 100% in LME; anisoperistaltic anastomosis 69% in EME and 62% in LME) respectively. Three studies [22–24] reported surgical POR and were included in the quantitative analysis. These studies did not report significant differences in postoperative pharmacological prophylaxis or in smoking between the EME and LME subgroups. Concerning preoperative treatment, Coffey et al. [22] included more patients with biologics in EME group (44% vs. 17%, *p* = 0.043), while in Mineccia et al. [24] the steroids administration was higher in LME (27.9% vs. 13.7%). Overall POC rates could be extracted from three studies [23, 24, 39], anastomotic leak from four studies [23, 37–39], reoperation from three studies [23, 37, 39], surgical site infection (SSI) from three studies [23, 37, 39], hospitalization from four studies [23, 24, 37, 38], and operative time from three studies [24, 37, 38]. Ewe et al. reported recurrence-free time interval [36], and Mineccia et al. [24] and de Willebois et al. [38] explored endoscopic recurrence, results that were presented in the qualitative analysis.

**Table 1:** Main characteristics of the included studies

	Author	Country	Study design	Study period	Sample size (female%)	Intestinal resection type	Age at resection	Extended mesenteric excision	Limited mesenteric excision	Follow-up period	Outcome
Quantitative analysis	van der Does de Willebois [38] (2024)	Netherlands, Italy	Randomised controlled trial, six centers (two in Italy, four in Netherlands)	2020–2023	133 (57.14%) 67 EME 66 LME	Ileocolic resection	36 (27–58) (EME) 37 (24–48 (LME) (median (IQR), years)	Mesentery divided up to the level of the ileocolic trunk, following the lower border of the ileocolic artery	Mesentery divided close to the bowel wall	6 months	Endoscopic recurrence at 6 months; anastomotic leak; hospitalization; operative time; Clavien–Dindo; blood loss
	Abdulkarim [37] (2023)	USA, Canada	Cohort, retrospective, ACS-NSQIP database (over 600 centers)	2014–2019	3709 (53.97%) 622 EME 3087 LME	Segmental colectomy	41 ± 15.78 (EME) 42.3 ± 16.76 (LME) (mean ± SD, years)	Lymph node yield (≥ 12 lymph nodes) mean was 17.89, SD 7.74)	Lymph node yield (< 12 lymph nodes, mean was 3.86, SD 3.11)	30 days	Major morbidity; surgical site infection; anastomotic leak; reoperation; hospitalization; operative time; bleeding, other ( e.g., dehiscence, sepsis, septic shock, abdominal complications, death, bleeding, pneumonia, thromboembolic events, acute renal failure, urinary tract infection, cerebrovascular accident, cardiac arrest, myocardial infarction)
	Mineccia [24] (2022)	Italy	Cohort, retrospective, two centers (different intervention for each center)	2009–2019	326 (41.41%) 204 EME 122 LME	Ileocolic resection	40.5 ± 14.7 (EME) 40.7 ± 16 (LME) (mean ± SD, years)	Ligation of the vessel at D2 level, dissection of the mesentery and lymph nodes	Mesentery divided flush with the intestine	4.7 ± 3 (median, years)	Overall POC/ Clavien–Dindo; endoscopic recurrence; ultrasound recurrence; surgical recurrence; hospitalization
	Zhu [23] (2021)	China	Cohort, retrospective, single center	2000–2018	126 (29.36%) 66 EME 60 LME	Colorectal resection	30.42 ± 10.15 (EME) 31.15 ± 10.36 (LME) (mean ± SD, years)	Mesentery divided 1-cm distant from the origin of major arterial trunks	Mesentery divided close to the bowel wall	47.5 ± 23.67 (EME) 45.12 ± 25.45 (LME) (mean ± SD, months)	Overall POC; surgical site infection; anastomotic leak; reoperation; surgical recurrence; hospitalization; other (dysfunction GI recovery, intraabdominal bleeding/abscess, blood loss)
	Coffey [22, 39] (2018)	Ireland	Cohort, retrospective (LME) and prospective (EME), single center	2004–2010 (LME) after 2010 (EME)	64 (56.25%) 34 EME 30 LME	Ileocolic resection	35.9 ± 11.87 (EME) 37.7 ± 13.67 (LME) (mean ± SD, years)	Mesentery fully detached and divided close to the mesenteric root	Mesentery divided flush with the intestine	51.7 ± 20.98 (EME) 69.9 ± 48.47 (LME) (mean ± SD, months)	Overall POC; surgical site infection; anastomotic leak; reoperation; surgical recurrence; other (sepsis, intraabdominal collection, drainage, abdominal wound revision)
Qualitative analysis	Ewe [36] (1989)	Germany	RCT (non-randomized for surgical intervention), multicentric	N/A	232 (51.29%) 86 EME 146 LME	Intestinal resections	31 (15–66) (median (range), years)	Radical operation (≥ 10 cm of non-involved proximal and distal bowel segments; lymph nodes removed as in cancer procedure)	Non-radical operation (< 10 cm of uninvolved bowel; lymph node dissection adjacent to the gut)	3 years	Overall postoperative recurrence (radiologic, endoscopic or surgical recurrence)

*ACS-NSQIP* American College of Surgeons National Surgical Improvement Program, *D2* descending part of duodenum, *EME* extended mesenteric excision, *IQR* interquartile range, *LME* limited mesenteric excision, *N/A* not available, *POC* postoperative complications, *RCT* randomized controlled trial, *SD* standard deviation

### Quantitative synthesis

#### Surgical recurrence

**Fig. 2:**
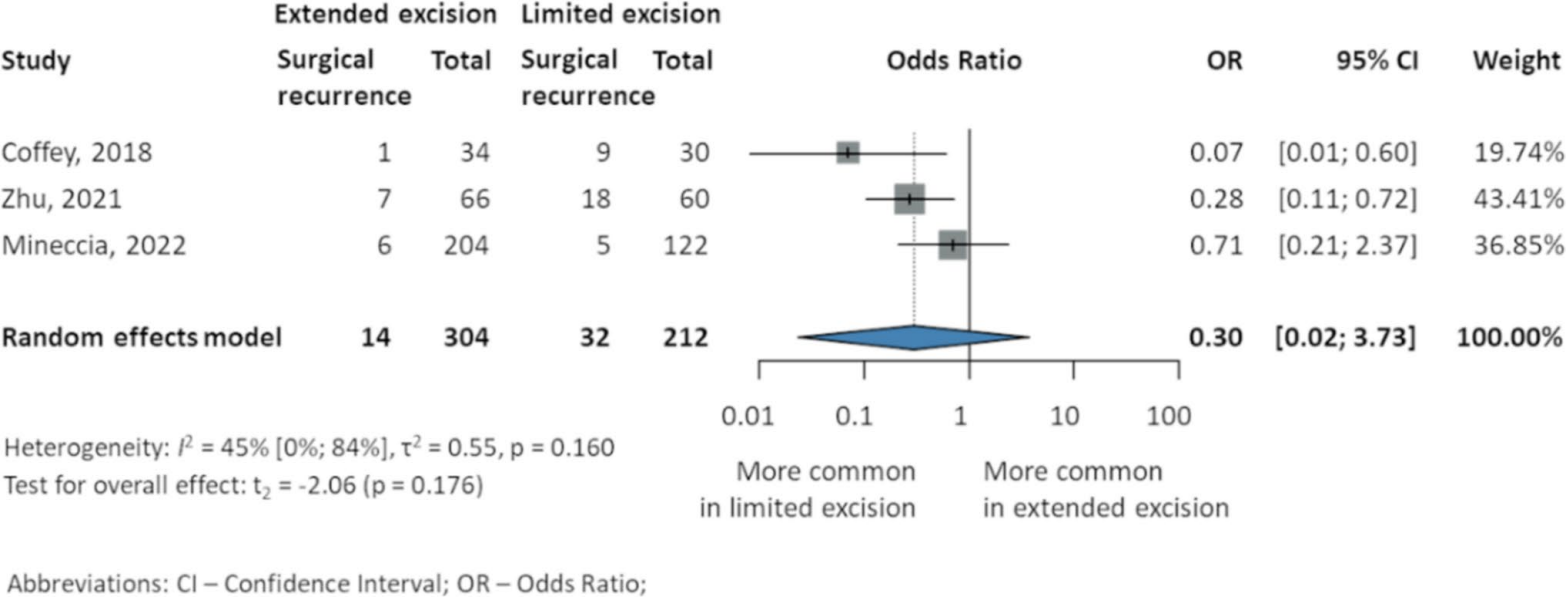
Postoperative surgical recurrence in patients with Crohn’s disease after intestinal resection

Pooled data from three studies [22–24] analyzing 516 patients showed no significant difference regarding surgical recurrence (OR 0.3, 95% CI 0.02–3.73; *p* = 0.176; *I*^2^ 45% 95% CI 0–84%), although lower rates in EME were revealed in each study: Coffey et al. [22] (1 EME, 2.9%, 51.7 ± 20.98 months follow-up vs. 9 LME, 30%, 69.9 ± 48.47 months), Zhu et al. [23] (7 EME, 10.6%, 47.5 ± 23.67 months vs. 18 LME, 30%, 45.12 ± 25.45 months), and Mineccia et al. [24] (5-year estimate, 6 EME, 2.8% vs. 5 LME, 4%) (Fig. 2).

#### Postoperative complications

**Fig. 3:**
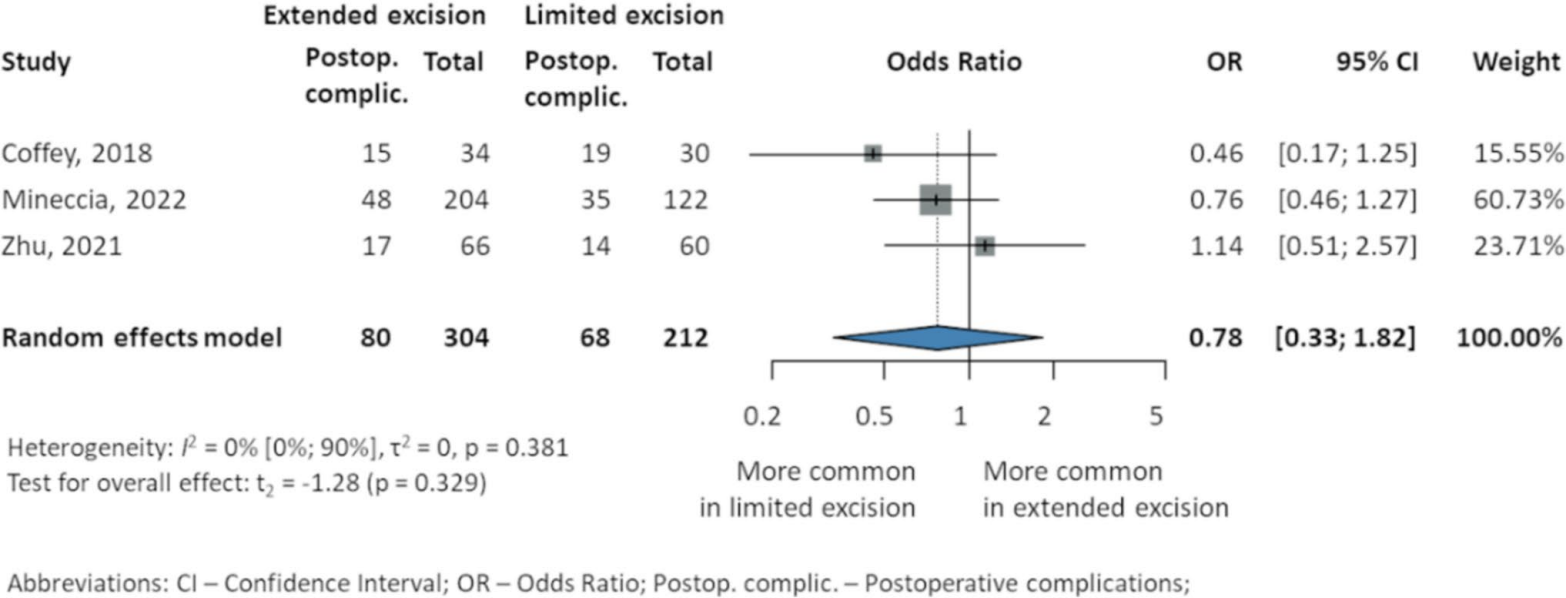
Overall postoperative complications in patients with Crohn’s disease after intestinal resection

The overall rate of POC reported in three studies [23, 24, 39] (304 EME, 212 LME) revealed no significant difference between EME and LME (OR 0.78, 95% CI 0.33–1.82; *p* = 0.329; *I*^2^ 0% 95% CI 0–90%). Two studies suggested lower rates of POC in EME compared to LME, Coffey et al. [39] (15 EME, 44% vs. 19 LME, 63%), and Mineccia et al. [24] (48 EME, 23.5% vs. 35 LME, 28.6%), while Zhu et al. [23] reported higher rates (17 EME, 25.8% vs. 14 LME, 23.3%) (Fig. 3).

### Anastomotic leak

**Fig. 4:**
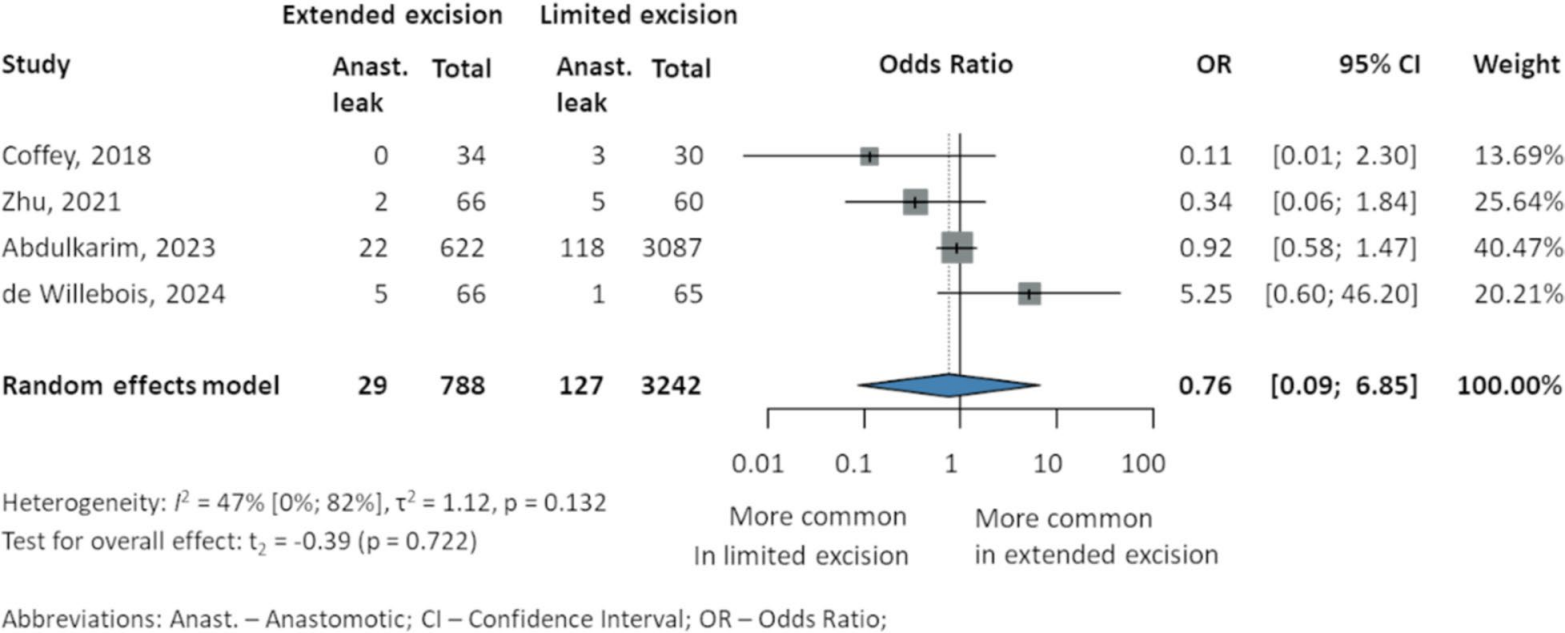
Anastomotic leak in patients with Crohn’s disease after intestinal resection

Four studies [23, 37–39] including 4030 patients reported anastomotic leak, showing no significant differences between the subgroups: Coffey et al. [39] (0 EME vs. 3 LME, 10%), Zhu et al. [23] (2 EME, 3% vs. 5 LME, 8.3%), Abdulkarim et al. [37] (22 EME, 3.54% vs. 118 LME, 3.82%), and de Willebois et al. [38] (5 EME, 8% vs. 1 LME, 2%). Overall effect was not statistically significant (OR 0.76, 95% CI 0.09–6.85, *p* = 0.722; *I*^2^ 47% 95% CI 0–82%) (Fig. 4).

### Surgical site infection

**Fig. 5:**
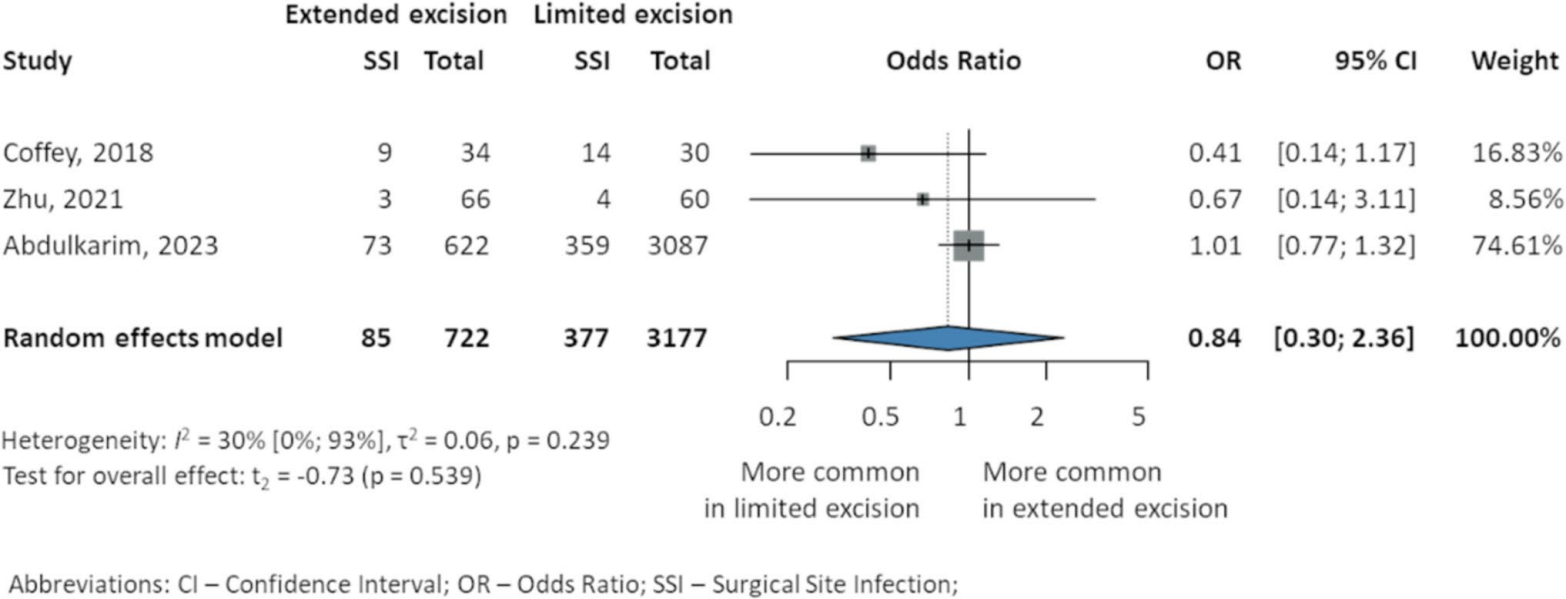
Surgical site infection in patients with Crohn’s disease after intestinal resection

The SSI rates were not statistically different in the results presented by Coffey et al. [39] (9 EME, 27% vs. 14 LME, 47%), Zhu et al. [23] (3 EME, 4.5% vs. 4 LME, 6.6%), and Abdulkarim et al. [37] (73 EME, 11.74% vs. 359 LME, 11.63%). The overall analysis of the 3899 patients showed nonsignificant results (OR 0.84, 95% CI 0.3–2.36, *p* = 0.539; *I*^2^ 30% 95% CI 0–93%) (Fig. 5).

### Reoperation

**Fig. 6:**
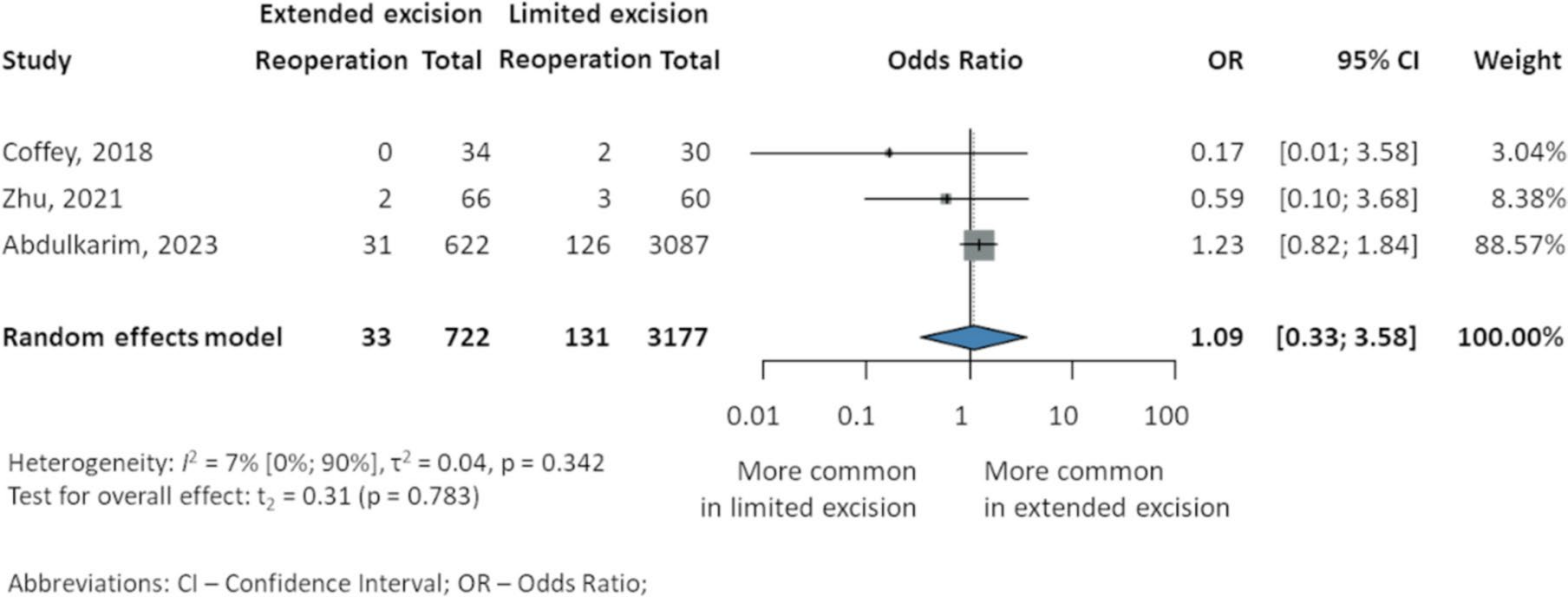
Reoperation in patients with Crohn’s disease after intestinal resection

No significant differences were reported overall reoperation rates (OR 1.09, 95% CI 0.33–3.58, *p* = 0.783* I*^2^ 7% 95% CI 0–90%). Three studies [23, 37, 39], including 3899 patients, reported reinterventions, without any statistically significant difference: Coffey et al. [39] (0 EME, 0% vs. 2 LME, 7%), Zhu et al. [23] (2 EME, 3% vs. 3 LME, 5%), and Abdulkarim et al. [37] (31 EME, 4.98% vs. 126 LME, 4.08%) (Fig. 6).

### Length of hospital stay

**Fig. 7:**
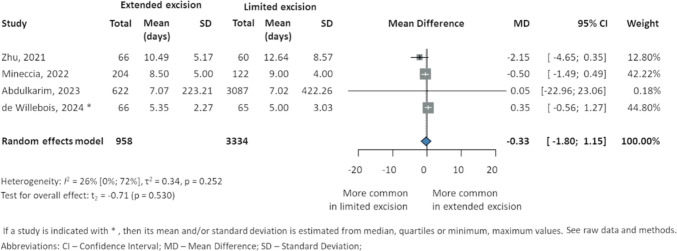
Postoperative length of hospital stay in patients with Crohn’s disease after intestinal resection

The postoperative hospital stay was similar between the groups in all four studies [23, 24, 37, 38]: Mineccia et al. [24] (8.5 ± 5 days EME vs. 9 ± 4 days LME), Zhu et al. [23] (10.49 ± 5.17 days EME vs. 12.64 ± 8.57 days LME), Abdulkarim et al. [37] (7.07 (SE 8.95) days EME vs. 7.02 (SE 7.6) days LME), and de Willebois et al. [38] (5 (IQR 4–7) days EME vs. 5 (IQR 3–7) days LME). The pooled data of postoperative hospitalization, covering a total of 4292 patients, found a MD of − 0.33 (95% CI − 1.8 to 1.15, *p* = 0.530) (Fig. 7).

### Operative time

**Fig. 8:**
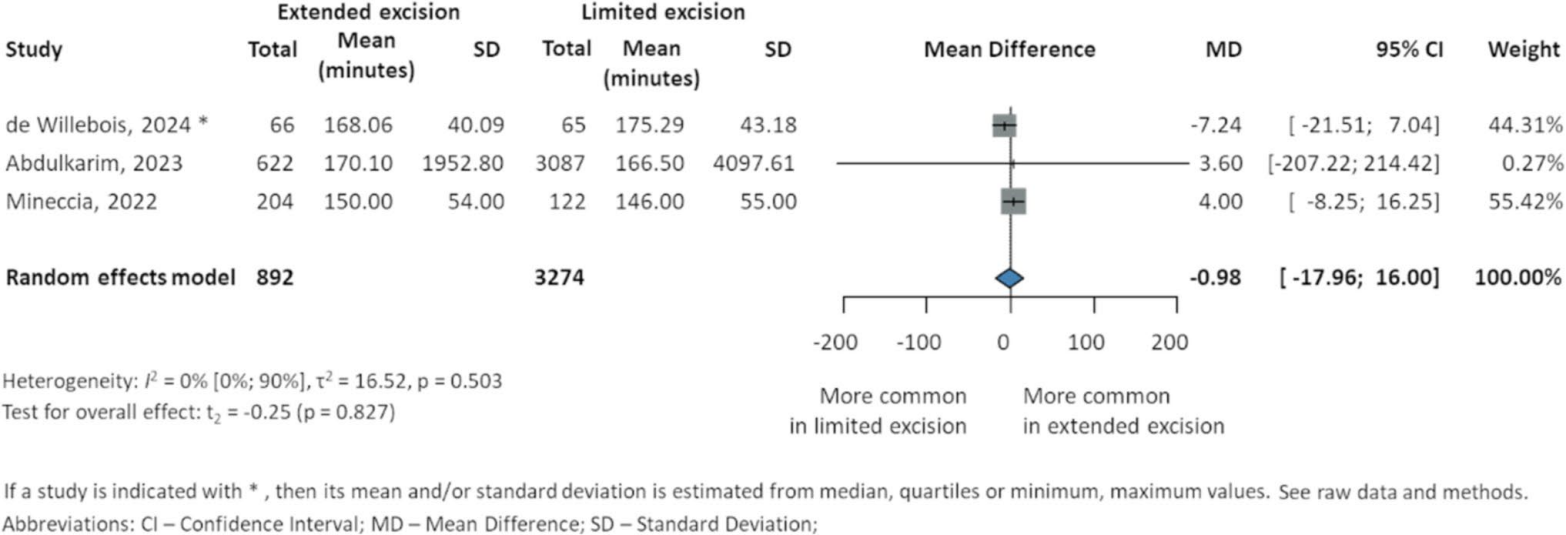
Operative time in patients with Crohn’s disease during intestinal resection

The overall MD for operative time reported in three studies [24, 37, 38] was − 0.98 (95% CI − 17.96 to 16, *p* = 0.827). Individual studies reported nonsignificant differences: Mineccia et al. [24] (150 ± 54 min EME vs. 146 ± 55 min LME), Abdulkarim et al. [37] (170.1(SE 78.3) min EME vs. 166.5 (SE 73.75) min LME), and de Willebois et al. [38] (2 h 47 min (IQR 2 h 22min–3 h 15 min) EME vs. 2 h 50 min (IQR 2 h 29 min–3 h 26 min) LME) (Fig. 8).

### Qualitative synthesis

#### Postoperative recurrence

##### Postoperative recurrence-free interval

Ewe et al. [36] analyzed 232 patients with intestinal resection who had a radical (86 patients, 37%) or non-radical operation (146 patients, 63%) and who were further randomized to prophylactic sulfasalazine treatment (111 patients, 48%) or placebo (121 patients, 52%). When radiologic, endoscopic, or surgical recurrence-free intervals were compared, longer periods were reported for the non-radical subgroup in both placebo (mean ± SE 29.4 ± 1.2 vs. 19.5 ± 2.4 months) and sulfasalazine (mean ± SE 34.4 ± 1.6 vs. 22.3 ± 2.5 months) arms.

##### Endoscopic recurrence

Using the modified Rutgeerts score, de Willebois et al. analyzed the endoscopic recurrence (≥ i2b) at 6 months follow-up. Recurrence was present in  28 EME patients (42%) and 28 LME (43%), but the results were not statistically significant (RR 0.985, 95% CI 0.663–1.464; *p* = 1). No significant differences were found regarding the severity of the lesions.

Mineccia et al. [24] evaluated patients between 6 and 12 months after surgery and assessed endoscopic findings using the Rutgeerts score, but found no significant differences. A score between i2 and i4 was reported in 91 EME patients (44.6%) and 57 LME (46.7%) during follow-up (*p* = 0.7), indicating endoscopic recurrence.

##### Ultrasonographic recurrence

Minnecia et al. [24] also evaluated the ultrasonographic recurrence between 6 and 12 months follow-up for 290 patients of 326, but no significant differences were found between the two types of intestinal resection (40.4% vs. 41.4%).

#### Postoperative complications

Abdulkarim et al. [37] evaluated 30-day postoperative morbidity as major morbidity, represented by a composite of different complications, that was similar in both groups (91 EME patients, 14.63% vs. 442 LME, 14.32%). There were no significant differences regarding overall abdominal complications (131 EME, 21.06% vs. 711 LME, 23.03%), or other specific complications (superficial SSI, deep SSI, organ space SSI, dehiscence, bleeding requiring transfusion, sepsis, septic shock, pneumonia, thrombotic complications, acute renal failure, urinary tract infection, cerebrovascular accident, cardiac arrest, myocardial infarction, and death).

Regarding intraoperative blood loss, the results were similar between EME and LME in the study of de Willebois et al. [38], while significant differences were reported by Zhu et al. [23] (83.1 ± 56.19 vs. 132.25 ± 85.53, *p* = 0.002).

Mineccia et al. [24] and de Willebois et al. [38] used Clavien–Dindo classification to report the POC, and both studies showed no significant differences between subgroups. Thus, Mineccia et al. [24] reported grade I complications (11 EME, 5.4% vs. 7 LME, 5.7%), grade II (20 EME, 9.8% vs. 16 LME, 13.1%), grade III (16 EME, 7.8% vs. 11 LME, 9%), and grade IV (1 EME, 0.5% vs. 1 LME, 0.8%), respectively. De Willebois et al. [38] reported only complications grade ≥ IIIa: grade IIIa (2 LME, 3%), grade IIIb (6 EME, 9% vs. 3 LME, 5%), and grade IV (1 EME, 2%). There were no grade V complications in any of the two studies.

Zhu et al. [23] identified similar POC rates in EME and LME for intra-abdominal abscess (2 EME, 3%), ileus (2 EME, 3%), dysfunctional of gastrointestinal recovery (6 EME, 9% vs. 2 LME, 3.3%), and intra-abdominal bleeding (2 LME, 3.3%).

Similar POC were also reported by Coffey et al. [22, 39] when comparing EME with LME, including intra-abdominal/pelvic collection (3 EME, 9% vs. 5 LME, 17%), sepsis (3 LME, 10%), collection drainage (3 LME, 10%), and revision of abdominal wound (2 LME, 7%), no deaths being recorded.

### Risk of bias assessment

The assessment is visualized in the Supplementary Material (Figs. S1–S14). For the outcomes included in the meta-analysis, the studies predominantly associated a moderate level of bias due to possible interference of confounding factors and questionable standardization when reporting surgical recurrence as an outcome. The indication for surgery depends on the expertise of the medical team and might be subjective. Low and moderate levels of bias were reported for endoscopic recurrence and hospitalization, respectively. One study included solely in the qualitative synthesis showed a high level of bias, in terms of heterogeneity in assessing outcomes, selective reporting of the results, or lacking information on possible confounders and cointerventions.

### Certainty of evidence

The levels for the pooled outcomes were very low and low, as shown in the Supplementary Material. Although there were some concerns regarding the risk of bias, the inconsistency and imprecision of the results considerably downgraded the quality of evidence (Table S3).

## Discussion

We assessed the association between mesenterectomy during intestinal resections and postsurgical outcomes in patients with CD. Our meta-analytical calculations did not show any statistically significant difference between EME and LME regarding long-term surgical recurrence or short-term POC, including overall POC, anastomotic leak, SSI, reoperation, or hospitalization. In terms of endoscopic recurrence, ultrasonographic recurrence, or other postoperative complications, individual studies reported similar results between the two groups.

Current guidelines state that the length of bowel resections should be approached in a conservative manner [12, 15, 16]. Conventionally, a division of the mesentery close to the intestinal wall is preferred, one reason being the fear of surgical complications. A hypertrophic mesentery might carry a high risk of bleeding due to friability, hypervascularization, and difficulty in differentiating vascular from avascular areas [14]. Moreover, central ligation of the main vessels might affect the blood flow in the intestine and, therefore, preservation of the mesentery could provide a better blood supply.

The mesentery drew attention in 2016, when Coffey et al. categorized it as an organ [40]. Currently, its full pathophysiological mechanisms and functions are still under investigation. Mesentery-based surgical techniques have already proved their effectiveness in surgical oncology, and Coffey et al. have speculated that including the mesentery in CD intestinal resection would improve the recurrence rate. However, the assumptions were made on the basis of the results of a small cohort study that they published in 2018, whose reliability is limited [14, 41, 42]. Since there were no clear recommendations regarding the extent and technique of mesenterectomy, Willebois et al. proposed a standardization for laparoscopic EME in ileocolic resection (ICR) for CD in 2020 [43].

Studies have shown that the mesentery is actively involved in the pathological processes of CD. Bacterial translocation is incriminated in CrF development and the mesentery might restrain the systemic dissemination of bacterial antigens [44–46]. The mesenteric adipose tissue modulates local hormonal and immune homeostasis through adipokines secretion, both pro-inflammatory (e.g., leptin, and resistin) and anti-inflammatory molecules (e.g., adiponectin, and ghrelin) being released [47–49], and is also involved in C-reactive protein synthesis [44]. Moreover, CrF monocyte differentiation predominantly into M2 macrophages favors collagen synthesis and fibrogenesis [47–49].

The mesorectum was also investigated, although it is spared from macroscopical changes and the pathophysiological mechanisms might differ. De Groof et al. revealed that in patients with CD, total mesorectal excision during proctectomy was associated with fewer postoperative perineal complications and higher healing rates than limited excision. Moreover, the mesorectum expressed pro-inflammatory immune markers. Interestingly, no significant differences were reported in the ulcerative colitis group [50].

Mesenteric alterations and their severity were connected to mucosal damage in the study of Coffey et al. [22]; however, the heterogeneity of mesenteric lesions is still not fully understood. CrF frequently associates with fibrostenoses [51–53]. Sampietro et al. showed that thickened mesentery was associated with penetrating behavior, surgery for strictures, small bowel locations and resections [54]. Recently, Bislenghi et al. indicated no association between 12 months POR and the macroscopic aspect of the intestine and mesentery during surgery [55].

Stricturoplasty represents a viable option for small bowel fibrostenoses in CD, sparing the bowel and leaving the mesentery on site. Although a meta-analysis of 1026 patients published by Butt et al. indicated higher risk of disease recurrence and shorter recurrence-free survival interval in stricturoplasty compared to bowel resection [56], recent studies reported similar rates of POR [57, 58], contrary to the hypothesized effect of the mesentery.

All studies included in our meta-analysis reported lower odds of surgical recurrence when removing the mesentery. Coffey et al. [22] showed a strong association between mesentery preservation and surgical recurrence; however, this study has several drawbacks, apart from the small number of patients. The authors compared a prospective cohort who underwent EME with a historical one with LME, extracted from a prospectively maintained database. The different time frames could be a source of confounding due to dissimilarities in therapeutic options, local protocols, and guideline recommendations. Moreover, although 92% of the relapses occurred in the first 24 months, the follow-up period was longer in the control group.

Including studies with patients from different periods could influence the overall POR rates, as the continuous improvement of CD management reduced the need for surgical reintervention in recent years [10]. This could contribute to the low recurrence reported by Mineccia et al. [24], which included patients from 2009 to 2019. The study by Ewe et al. [36] showed higher rates of POR and shorter relapse-free intervals in the radical resection group, but should be interpreted cautiously as it was published more than 30 years ago, the extent of mesenterectomy was not exactly specified, the relapse was defined as a composite of radiologic, endoscopic, or surgical recurrence, and proper prophylactic therapy was lacking.

Despite the fact that the EME group showed a reduced OR for overall POC in two studies [24, 39], these findings contradict the results of Zhu et al. [23]. We cannot have a comprehensive interpretation of the results because of insufficient information on possible confounders. Some important factors were not reported in all studies, including the timing of surgery, preoperative optimization, and indication for surgery across subgroups. Moreover, as data were reported from single centers, postoperative morbidity could depend on the expertise of the medical team at each hospital.

Interpreting data from the Abdulkarim et al. [37] study is challenging, since several baseline details were not provided. De Willebois et al. [38] reported a trend to higher rates of anastomotic leak in the EME group; however, the overall rate was similar to that reported in the literature. We have to consider that the training was done by one surgeon using a video vignette; hence, the experience in EME might have varied between surgeons. Also, surgery indication is missing and there were numerically more patients with preoperative biologics in the EME (52% vs. 38%).

The results of the first RCT published by de Willebois et al. [38] indicated similar rates of endoscopic recurrence between groups at 6 months. However, the follow-up period is short and also differences regarding some possible confounders were noted, although not statistically significant, such as age of onset, perianal involvement, preoperative and postoperative medication, and anastomotic leak. Long-term results are necessary to adequately evaluate the impact of the mesentery resection on the CD outcome.

### Strengths and limitations

The role of mesentery in CD management is an emergent concept that has recently started being explored in depth. Our study represents a comprehensive review that also included meta-analytical calculations.

Our study has some limitations. The information was scarce; thus, the meta-analyses comprised only few studies that analyzed both ileocolic and colorectal resections. We could not pool data for clinical, endoscopic, or radiologic recurrence. We included only one RCT and, although the observational studies reported data mainly from post hoc analyses of prospectively maintained databases, the confounding factors could not be adequately controlled. As the study number is highly limited, the estimation of heterogeneity is very uncertain and the meta-analysis provided a low and very low certainty of evidence.

### Implications for practice and research

Neither basic nor clinical research studies have so far offered sufficient information to sustain management recommendations in CD [59, 60]. Multicentric, large-sample RCTs are needed to limit the confounding factor interference and also to evaluate the impact on clinical, endoscopic, and radiologic recurrence, in order to clarify the impact on long-term outcomes. As the development of CrF is described especially in the small bowel, resections of specific anatomic intestinal segments should be investigated. Moreover, in surgical practice, partial mesenterectomy might sometimes be performed, and should be taken into account. Fundamental insights into microbiome involvement and immuno-hormonal mechanisms have yet to be deciphered and whether these pathways could become targets for novel therapeutics should be investigated.

Several trials are currently ongoing (NCT04538638, NCT03769922, NCT04539665, NCT04578392, NCT02542904). Results from the ongoing MEErKAT British RCT (ISRCTN16900055) that analyzes both the mesenterectomy (radical vs. close) and the anastomosis type (Kono-S vs. standard) are expected.

## Conclusion

Our results do not support the superiority of performing intestinal resections with extended mesenteric excision compared to limited excision in terms of short-term postoperative complications and long-term postoperative recurrence, considering that the statistical significance was not reached. In the context of the limited study number, the interpretation of the results requires a cautious approach and strong conclusions could not be drawn.

## Supplementary Information

Below is the link to the electronic supplementary material.

Supplementary file1 (DOCX 1310 KB)

## Data Availability

The datasets used in this study can be found in the full-text articles included in the systematic review and meta-analysis.
