# Supplementary material for: To cut or not to cut? Extended mesenteric excision during intestinal resection does not impact the postoperative recurrence nor the postoperative complications in Crohn’s disease: a systematic review and meta-analysis
Source: Tech Coloproctol. 2025 Mar 8;29(1):79. doi: 10.1007/s10151-025-03110-w (PMC11890256; doi:10.1007/s10151-025-03110-w)
Supplement: Supplementary file 1 — Supplementary file1 (DOCX 1310 KB) [file 10151_2025_3110_MOESM1_ESM.docx]

Supplementary Material

**To cut or not to cut? Extended mesenteric excision during intestinal resection does not impact the postoperative recurrence nor the postoperative complications in Crohn’s disease: a systematic review and meta-analysis**

**Authors:**

Mihaela Topala^1,2^, MD; Petrana Martinekova^2,3^, MD; Anett Rancz^2,4^, MD; Dániel Sándor Veres^2,5^, PhD; Katalin Lenti^6^, PhD; Pál Miheller^7^, MD, PhD; Bálint Erőss^2,8,9^, MD, PhD; Péter Hegyi^2,8,9^, MD, PhD, DSc; Szabolcs Ábrahám^2,10^, MD, PhD

**Affiliations:**

1. Carol Davila University of Medicine and Pharmacy, Bucharest, Romania
2. Centre For Translational Medicine, Semmelweis University, Budapest, Hungary
3. EDU Institute of Higher Education, Medicine and Health, Kalkara, Malta
4. Department of Internal Medicine and Hematology, Medical School, Semmelweis University, Budapest, Hungary
5. Department of Biophysics and Radiation Biology, Semmelweis University, Budapest, Hungary
6. Department of Morphology and Physiology, Faculty of Health Sciences, Semmelweis University, Budapest, Hungary
7. Department of Surgery, Transplantation and Gastroenterology, Semmelweis University, Budapest, Hungary
8. Institute for Translational Medicine, Medical School, University of Pécs, Pecs, Hungary
9. Institute of Pancreatic Diseases, Semmelweis University, Budapest, Hungary
10. Department of Surgery, Faculty of Medicine, University of Szeged, Szeged, Hungary

**Corresponding author**

Szabolcs Ábrahám MD, PhD

Centre For Translational Medicine, Semmelweis University, Budapest, Hungary

Postal address: 1085 Budapest, Üllői út 26, Hungary

Tel.: +36 70 580 1766

E-mail address: [rancz.anett@semmelweis.hu](mailto:rancz.anett@semmelweis.hu)

ORCID: 0000-0002-2191-1714

**Table S1.** PRISMA checklist

| **Section and Topic** | **Item #** | **Checklist item** | **Location where item is reported** |
| --- | --- | --- | --- |
| **TITLE** | | |  |
| Title | 1 | Identify the report as a systematic review. | 1 |
| **ABSTRACT** | | |  |
| Abstract | 2 | See the PRISMA 2020 for Abstracts checklist. | 1 |
| **INTRODUCTION** | | |  |
| Rationale | 3 | Describe the rationale for the review in the context of existing knowledge. | 2 |
| Objectives | 4 | Provide an explicit statement of the objective(s) or question(s) the review addresses. | 2 |
| **METHODS** | | |  |
| Eligibility criteria | 5 | Specify the inclusion and exclusion criteria for the review and how studies were grouped for the syntheses. | 2 |
| Information sources | 6 | Specify all databases, registers, websites, organisations, reference lists and other sources searched or consulted to identify studies. Specify the date when each source was last searched or consulted. | 2 |
| Search strategy | 7 | Present the full search strategies for all databases, registers and websites, including any filters and limits used. | 2  Supplem. 4 |
| Selection process | 8 | Specify the methods used to decide whether a study met the inclusion criteria of the review, including how many reviewers screened each record and each report retrieved, whether they worked independently, and if applicable, details of automation tools used in the process. | 3 |
| Data collection process | 9 | Specify the methods used to collect data from reports, including how many reviewers collected data from each report, whether they worked independently, any processes for obtaining or confirming data from study investigators, and if applicable, details of automation tools used in the process. | 3 |
| Data items | 10a | List and define all outcomes for which data were sought. Specify whether all results that were compatible with each outcome domain in each study were sought (e.g. for all measures, time points, analyses), and if not, the methods used to decide which results to collect. | 3  Supplem. 5 |
|  | 10b | List and define all other variables for which data were sought (e.g. participant and intervention characteristics, funding sources). Describe any assumptions made about any missing or unclear information. | 3  Supplem. 5 |
| Study risk of bias assessment | 11 | Specify the methods used to assess risk of bias in the included studies, including details of the tool(s) used, how many reviewers assessed each study and whether they worked independently, and if applicable, details of automation tools used in the process. | 3  Supplem. 5 |
| Effect measures | 12 | Specify for each outcome the effect measure(s) (e.g. risk ratio, mean difference) used in the synthesis or presentation of results. | 3  Supplem. 5, 6 |
| Synthesis methods | 13a | Describe the processes used to decide which studies were eligible for each synthesis (e.g. tabulating the study intervention characteristics and comparing against the planned groups for each synthesis (item #5)). | 3  Supplem. 5, 6 |
|  | 13b | Describe any methods required to prepare the data for presentation or synthesis, such as handling of missing summary statistics, or data conversions. | Supplem. 5, 6 |
|  | 13c | Describe any methods used to tabulate or visually display results of individual studies and syntheses. | 3  Supplem. 5, 6 |
|  | 13d | Describe any methods used to synthesize results and provide a rationale for the choice(s). If meta-analysis was performed, describe the model(s), method(s) to identify the presence and extent of statistical heterogeneity, and software package(s) used. | 3  Supplem. 5, 6 |
|  | 13e | Describe any methods used to explore possible causes of heterogeneity among study results (e.g. subgroup analysis, meta-regression). | Supplem. 5, 6 |
|  | 13f | Describe any sensitivity analyses conducted to assess robustness of the synthesized results. | N/A |
| Reporting bias assessment | 14 | Describe any methods used to assess risk of bias due to missing results in a synthesis (arising from reporting biases). | 3  Supplem. 5 |
| Certainty assessment | 15 | Describe any methods used to assess certainty (or confidence) in the body of evidence for an outcome. | 3 |
| **RESULTS** | | |  |
| Study selection | 16a | Describe the results of the search and selection process, from the number of records identified in the search to the number of studies included in the review, ideally using a flow diagram. | 3, 4 |
|  | 16b | Cite studies that might appear to meet the inclusion criteria, but which were excluded, and explain why they were excluded. | 4 |
| Study characteristics | 17 | Cite each included study and present its characteristics. | 3-7  Supplem. 7-8 |
| Risk of bias in studies | 18 | Present assessments of risk of bias for each included study. | 11  Supplem. 9-18 |
| Results of individual studies | 19 | For all outcomes, present, for each study: (a) summary statistics for each group (where appropriate) and (b) an effect estimate and its precision (e.g. confidence/credible interval), ideally using structured tables or plots. | 4, 7-11 |
| Results of syntheses | 20a | For each synthesis, briefly summarise the characteristics and risk of bias among contributing studies. | 11 |
|  | 20b | Present results of all statistical syntheses conducted. If meta-analysis was done, present for each the summary estimate and its precision (e.g. confidence/credible interval) and measures of statistical heterogeneity. If comparing groups, describe the direction of the effect. | 4, 7-10 |
|  | 20c | Present results of all investigations of possible causes of heterogeneity among study results. | N/A |
|  | 20d | Present results of all sensitivity analyses conducted to assess the robustness of the synthesized results. | N/A |
| Reporting biases | 21 | Present assessments of risk of bias due to missing results (arising from reporting biases) for each synthesis assessed. | 11  Supplem. 9-18 |
| Certainty of evidence | 22 | Present assessments of certainty (or confidence) in the body of evidence for each outcome assessed. | 11  Supplem. 19, 20 |
| **DISCUSSION** | | |  |
| Discussion | 23a | Provide a general interpretation of the results in the context of other evidence. | 11, 12 |
|  | 23b | Discuss any limitations of the evidence included in the review. | 12 |
|  | 23c | Discuss any limitations of the review processes used. | 12 |
|  | 23d | Discuss implications of the results for practice, policy, and future research. | 12, 13 |
| **OTHER INFORMATION** | | |  |
| Registration and protocol | 24a | Provide registration information for the review, including register name and registration number, or state that the review was not registered. | 2 |
|  | 24b | Indicate where the review protocol can be accessed, or state that a protocol was not prepared. | 2 |
|  | 24c | Describe and explain any amendments to information provided at registration or in the protocol. | 2 |
| Support | 25 | Describe sources of financial or non-financial support for the review, and the role of the funders or sponsors in the review. | 13 |
| Competing interests | 26 | Declare any competing interests of review authors. | 13 |
| Availability of data, code and other materials | 27 | Report which of the following are publicly available and where they can be found: template data collection forms; data extracted from included studies; data used for all analyses; analytic code; any other materials used in the review. | 13 |

Abbreviations: Supplem. – supplementary material; N/A - not available

*From:*  Page MJ, McKenzie JE, Bossuyt PM, Boutron I, Hoffmann TC, Mulrow CD, et al. The PRISMA 2020 statement: an updated guideline for reporting systematic reviews. BMJ 2021;372:n71. doi: 10.1136/bmj.n71. For more information, visit: <http://www.prisma-statement.org/>

**Search strategy**

Search key: (Crohn disease OR Crohn* OR inflammatory bowel disease OR IBD) AND (mesent* OR mesocolon*OR mesosigmoid*) AND (resect* OR excis* OR remov* OR exclu* OR inclu*)

1. PUBMED

((crohn disease[MeSH Terms]) OR (Crohn disease) OR (Crohn*) OR (inflammatory bowel disease) OR (IBD)) AND ((mesent*) OR (mesocolon*) OR (mesosigmoid*)) AND ((resect*) OR (excis*) OR (remov*) OR (exclu*) OR (inclu*))

2. WEB OF SCIENCE

(ALL=(crohn disease) OR ALL=(crohn) OR ALL=(inflammatory bowel disease) OR ALL=(IBD)) AND (ALL=(mesent*) OR ALL=(mesocolon*) OR ALL=(mesosigmoid*)) AND (ALL=(resect*) OR ALL=(excis*) OR ALL=(remov*) OR ALL=(exclu*) OR ALL=(inclu*))

3. SCOPUS

TITLE-ABS-KEY ( "Crohn's disease" OR crohn* OR "inflammatory bowel disease" OR ibd ) AND ( mesent* OR mesocolon* OR mesosigmoid* ) AND ( resect* OR excis* OR remov* OR exclu* OR inclu* )

4. CENTRAL

ID Search Hits

#1 MeSH descriptor: [Crohn Disease] explode all trees 1789

#2 Crohn's disease 5824

#3 Crohn* 6126

#4 inflammatory bowel disease 4753

#5 IBD 3107

#6 #1 OR #2 OR #3 OR #4 OR #5 9730

#7 mesent* 1329

#8 mesocolon* 53

#9 mesosigmoid* 2

#10 #7 OR #8 OR #9 1367

#11 resect* 32821

#12 excis* 8357

#13 remov* 45980

#14 exclu* 92225

#15 inclu* 627306

#16 #11 OR #12 OR #13 OR #14 OR #15 691641

#17 #6 AND #10 AND #16 81

5. EMBASE

('crohn disease'/exp OR 'crohn disease' OR (crohn AND ('disease'/exp OR disease)) OR 'crohn s disease' OR 'crohn*' OR 'inflammatory bowel disease'/exp OR 'inflammatory bowel disease' OR 'ibd') AND ('mesent*' OR 'mesocolon*' OR 'mesosigmoid*') AND ('resect*' OR 'excis*' OR 'remov*' OR 'exclu*' OR 'inclu*')

**Data Collection Process and Data Items**

The following information was extracted: publication details (title, author, publication year, countries, and Digital Object Identifier), methods (study design, study period, sample size, and follow-up period), population characteristics (age, sex, smoking status, age at diagnosis, disease duration, disease phenotype, preoperative and postoperative medical treatment), interventions (type of mesenterectomy), outcomes (postoperative recurrence, length of hospital stay, postoperative complications, and recurrence-free time interval). Additional information was requested from the authors, but no response was received.

**Risk of Bias Assessment**

Cohort studies were assessed with the Risk of Bias In Non-randomized Studies of Interventions (ROBINS-I) tool, through seven domains - confounding, selection, measurement classification of intervention, deviations from intended interventions, missing data, measurements of outcomes, and selection of the reported result.

For randomized controlled trials, we used the Revised Cochrane Risk of Bias Tool for Randomized Trials (RoB2), that evaluated five main domains: randomization process, deviations from intended interventions, missing outcome data, measurement of the outcome, and selection of the reported results.

**Synthesis Methods**

To calculate the study odds ratios (ORs) and the pooled OR for binary categorical outcomes, the total number of patients and the number of patients who developed the interested outcome were extracted in both groups (extended mesenteric excision (EME), limited mesenteric excision (LME)) separately. Although the included studies reported data on time-to-event for postoperative surgical recurrence, hazard ratios with confidence interval were not provided and it was not possible to accurately extract individual patient data from Kaplan-Meier curves. Therefore, to estimate the effect measures, the total number of patients and the number of patients who developed the event for each outcome were extracted from the text or from the Kaplan-Meier curves if only that was available. From the available data, the OR with 95% CI was estimated at 4 years of follow-up time (as the longest time with available data in all studies). We assumed that the number of censored data until this time was negligible. We reported the results as the odds of event in the EME group versus the odds of an event in the LME group. To calculate the mean differences (MDs) and the pooled MD for continuous outcomes, the sample size, the mean and standard deviation were extracted in both groups separately. If the standard deviation was not reported, but the standard error of the mean was available, we calculated the standard deviation by dividing the standard error by the square root of the sample size. In the case of the outcome of length of hospitalization and operative time, if the mean and standard deviation was not available, but the median and the quartiles were reported we extracted it and we estimated the mean and the standard deviation by the Luo[1] and Shi[2] methods. Based on our knowledge, these variables usually follow a lognormal distribution, there this estimation could not cause a bias. We reported the results as the mean in the EME group minus the mean in the LME group.

Pooled ORs were calculated using the Mantel-Haenszel method[3],[4]. Exact Mantel-Haenszel method (without continuity correction) was used to handle zero cell counts (as recommended by Cooper and Sweeting[5,6]). Inverse variance weighting method was used to calculate the pooled MD. We used a Hartung-Knapp adjustment[7,8] for CIs. This adjustment was applied only if it is more conservative then the classical one (as recommended in Jackson et al.[9] as hybrid method 2). To estimate the heterogeneity variance measure (τ2), the Paule-Mandel method[10] for categorical, and restricted maximum-likelihood estimator for continuous outcomes was used with the Q profile method for confidence interval (recommended in Harrer et al. and Veroniki et al.[11,12]). Due to the small number of studies, the prediction interval was not reported. The number of studies was not sufficient to perform subgroup analysis or to assess publication bias and the influential effect of individual studies on the pooled results.

References

1. Luo D, Wan X, Liu J, Tong T. Optimally estimating the sample mean from the sample size, median, mid-range, and/or mid-quartile range. Stat Methods Med Res [Internet]. 2018;27:1785–805. Available from: https://journals.sagepub.com/doi/10.1177/0962280216669183

2. Shi J, Luo D, Weng H, Zeng X, Lin L, Chu H, et al. Optimally estimating the sample standard deviation from the five‐number summary. Res Synth Methods [Internet]. 2020;11:641–54. Available from: https://onlinelibrary.wiley.com/doi/10.1002/jrsm.1429

3. Mantel N, Haenszel W. Statistical Aspects of the Analysis of Data From Retrospective Studies of Disease. JNCI J Natl Cancer Inst. 1959;22:719–48.

4. ROBINS J, GREENLAND S, BRESLOW NE. A general estimator for the variance of the Mantel-Haenszel odds ratio. Am J Epidemiol. 1986;124:719–23.

5. Cooper H, Hedges L, Valentine J. The handbook of research synthesis and meta-analysis. 2nd ed. New York: Russell Sage Foundation; 2009.

6. J. Sweeting M, J. Sutton A, C. Lambert P. What to add to nothing? Use and avoidance of continuity corrections in meta‐analysis of sparse data. Stat Med [Internet]. 2004;23:1351–75. Available from: https://onlinelibrary.wiley.com/doi/10.1002/sim.1761

7. Knapp G, Hartung J. Improved tests for a random effects meta-regression with a single covariate. Stat Med. 2003;22:2693–710.

8. IntHout J, Ioannidis JP, Borm GF. The Hartung-Knapp-Sidik-Jonkman method for random effects meta-analysis is straightforward and considerably outperforms the standard DerSimonian-Laird method. BMC Med Res Methodol. 2014;14:25.

9. Jackson D, Law M, Rücker G, Schwarzer G. The Hartung‐Knapp modification for random‐effects meta‐analysis: A useful refinement but are there any residual concerns? Stat Med [Internet]. 2017;36:3923–34. Available from: https://onlinelibrary.wiley.com/doi/10.1002/sim.7411

10. Paule RC, Mandel J. Consensus Values and Weighting Factors. J Res Natl Bur Stand (1934). 1982;87:377.

11. Harrer M, Cuijpers P, Ebert D. Doing Meta-Analysis With R: A Hands-On Guide. 1st ed. London: Chapman & Hall/CRC Press; 2021.

12. Veroniki AA, Jackson D, Viechtbauer W, Bender R, Bowden J, Knapp G, et al. Methods to estimate the between‐study variance and its uncertainty in meta‐analysis. Res Synth Methods. 2016;7:55–79.

**Table S2**. Main characteristics of the included patients

|  | Author | Sample size  female% | Age at diagnosis | Disease duration | Age at disease diagnosis | Disease location | Disease behaviour | Perianal disease | Smoking | Family history | EIM | Preoperative therapy | Postoperative therapy | Indication for surgery | Surgical access | Length of resection |
| --- | --- | --- | --- | --- | --- | --- | --- | --- | --- | --- | --- | --- | --- | --- | --- | --- |
| Quantitative analysis | van der Does de Willebois  (2024) | 133  57.14% | N/A | 53(21-102) EME  45(9-122) LME  (median, (IQR), months) | **≤16 years**  4 (6%) EME  8 (12%) LME  **17-40 years**  38 (57%) EME  40 (61%) LME  **>41 years**  25 (37%) EME  18 (27%) LME | **Terminal ileum**  44 (66%) EME  40 (61%) LME  **Ileocolonic**  23 (34%) EME  26 (39%) LME | **Inflammation**  22 (33%) EME  15 (23%) LME  **Stenosis**  28 (42%) EME  29 (44%) LME  **Penetrating**  17 (25%) EME  22 (33%) LME | 5 (7%) EME  12 (18%) LME | **Active**  15 (22%) EME  16 (24%) LME  **History**  15 (22%) EME  9 (14%) LME  **Nonsmoker**  37 (55%) EME  41 (62%) LME | N/A | N/A | **None**  24 (36%) EME  28 (42%) LME  **5-ASA**  0 (0%) EME  1 (2%) LME  **Thiopurines**  8 (12%) EME  10 (15%) LME  **Biologics**  35 (52%) EME  25 (38%) LME  **Small molecules**  0 (0%) EME  2 (3%) LME | **Prophylactic**  20 (30%) EME  - 18 biologics  - 1 mesalazine  - 1 thiopurine  12 (18%) LME  - 9 biologics  - 2 thiopurine  - 1 small mol.  **Therapeutic**  14 (21%) EME  - 13 biologics  - 1 thiopurine  22 (34%) LME  - 18 biologics  - 4 thiopurine | N/A | N/A | **Ileum**  19 (15-34) EME  25 (16-36) LME  (median (IQR), cm)  **Colon**  7 (6-9) EME  7 (5-8) LME  (median (IQR), cm) |
|  | Abdulkarim  (2023) | 3709  53.97% | N/A | N/A | N/A | N/A | N/A | N/A | 123 (19.8%) EME  697 (22.6%) LME | N/A | N/A | N/A | N/A | N/A | **Open**  216 (34.7%)  EME  1379 (44.7%) LME | N/A |
|  | Mineccia  (2022) | 326  41.41% | 33.1±13.8 EME  33.4±15.6 LME  (mean±SD, years) | 7.5±8.3 EME  7.7±8.6 LME  (mean±SD, years) | **≤16 years**  12 (5.9%) EME  15 (12.3%) LME  **17-40 years**  140 (68.6%) EME  68 (55.7%) LME  **>41 years**  52 (25.5%) EME  39 (32%) LME | **Terminal ileum**  - all included patients | **Stricturing**  67 (32.8%) EME  40 (32.8%) LME  **Penetrating**  137 (67.2%) EME  82 (67.2%) LME | 27 (13.2%) EME  16 (13.1%) LME | 75 (36.8%) EME  35 (28.7%) LME | 14 (6.9%) EME  4 (3.3%) LME | 19 (9.3%) EME  10 (8.2%) LME | **Washout/5-ASA**  114 (55.9%) EME  46 (37.7%) LME  **Steroids**  28 (13.7%) EME  34 (27.9%) LME  **ISx**  21 (10.3%) EME  13 (10.6%) LME  **Biologics**  32 (15.7%) EME  16 (13.1%) LME  **Combined therapy**  9 (4.4%) EME  13 (10.7%) LME | **ISx or Biologics**  117 (65%) EME  87 (59.5%) LME | **Stenosis**  169 (82.8%) EME  92 (75.4%) LME  **Abscess/ Fistula**  35 (17.2%) EME  30 (24.6%) LME | **Open**  14 (6.9%)  EME  32 (22.2%) LME  **Laparoscopic**  190 (93.1%) EME  90 (73.8%) LME | 24±14 EME  27±16 LME  (mean±SD, cm) |
|  | Zhu  (2021) | 126  29.36% | 25.2±9.7 EME  26.8±10.8 LME  (mean±SD, years) | 61.6±52.1 EME  51.1±49.3 LME  (mean±SD, month) | **≤16 years**  4 (6.1%) EME  7 (11.7%) LME  **17-40 years**  50 (75.8%) EME  44 (73.3%) LME  **>40 years**  12 (18.2%) EME  12 (15%) LME | **Colonic**  41 (62.1%) EME  32 (53.3%) LME  **Ileocolonic**  25 (37.9%) EME  28 (46.7%) LME  **Upper tract**  4 (6.1%) EME  1 (1.7%) LME | **Nonstricturing**  **Nonpenetrating**  3 (4.5%) EME  0 (0%) LME  **Stricturing**  39 (59.1%) EME  20 (33.3%) LME  **Penetrating**  24 (36.4%) EME  40 (66.7%) LME | 22 (33.3%) EME  25 (41.7%) LME | 5 (7.6%) EME  9 (15%) LME | N/A | N/A | **Steroids**  25 (37.9%) EME  21 (35%) LME  **ISx**  23 (34.8%) EME  19 (31.7%) LME  **Infliximab**  4 (6.3%) EME  4 (7.1%) LME | **ISx**  38 (57.6%) EME  29 (48.3%) LME  **Biologics**  4 (6.1%) EME  2 (3.3%) LME | N/A | N/A | N/A |
|  | Coffey  (2018) | 64  56.25% | 28±10.9 EME  30.3±11.9 LME  (mean±SD, years) | 70.7±78.8 EME  75±117.4 LME  (mean±SD, months) | **<40 years**  26 (76%) EME  23 (77%) LME  **≥40 years**  6 (18%) EME  6 (20%) LME  N/A  2 (6%) EME  1 (3%) LME | **Terminal ileum**  26 (76%) EME  23 (77%) LME  **Colonic**  0 (0%) EME  2 (6%) LME  **Ileocolonic**  6 (18%) EME  5 (17%) LME  **Upper tract**  2 (6%) EME  0 (0%) LME | **Nonstricturing**  **Nonpenetrating**  8 (24%) EME  16 (53%) LME  **Stricturing**  14 (41%) EME  6 (20%) LME  **Penetrating**  12 (35%) EME  8 (27%) LME | N/A | **Active**  18 (53%) EME  14 (47%) LME  **History**  2 (6%) EME  6 (20%)LME  **Nonsmoker**  13 (38%) EME  9 (30%) LME  **N/A**  1 (3%) EME  1 (3%) LME | 12 (35%) EME  8 (27%) LME | N/A | **Anti-inflammatory**  9 (27%) EME  15 (50%) LME  **Steroids**  12 (35%) EME  13 (43%) LME  **ISx**  10 (29%) EME  11 (37%) LME  **Biologicals**  15 (44%) EME  5 (17%) LME  **None**  5 (15%) EME  5 (17%) LME | **Imuran®**  3 (9%) EME  4 (13%) LME  **6-MP**  1 (3%) EME  0 (0%) LME  **Anti-TNF**  4 (12%) EME  2 (7%) LME  **None**  26 (76%) EME  19 (63%) LME | N/A | N/A | 28.6±10.99 EME  33.3±15.77 LME  (mean±SD, cm) |
| Qualitative analysis | Ewe  (1989) | 232  51.29% | N/A | N/A | N/A | **Terminal ileum**  2 (2%) EME  2 (1%) LME  **Colonic**  3 (4%) EME  11 (8%) LME  **Ileocolonic**  81 (94%) EME  133 (91%) LME | N/A | N/A | N/A | N/A | N/A | N/A | N/A | N/A | N/A | N/A |

Abbreviations: 6-MP - 6-mercaptopurine; anti-TNF - anti-tumor necrosis factor; cm – centimeters; EME – extended mesenteric excision; EIM – extraintestinal manifestations; ISx – immunosuppresants; LME – limited mesenteric excision; N/A - not available; SD – standard deviation;

**Figure S1**. Risk of bias assessment (ROBINS–I tool) for the surgical recurrence outcome
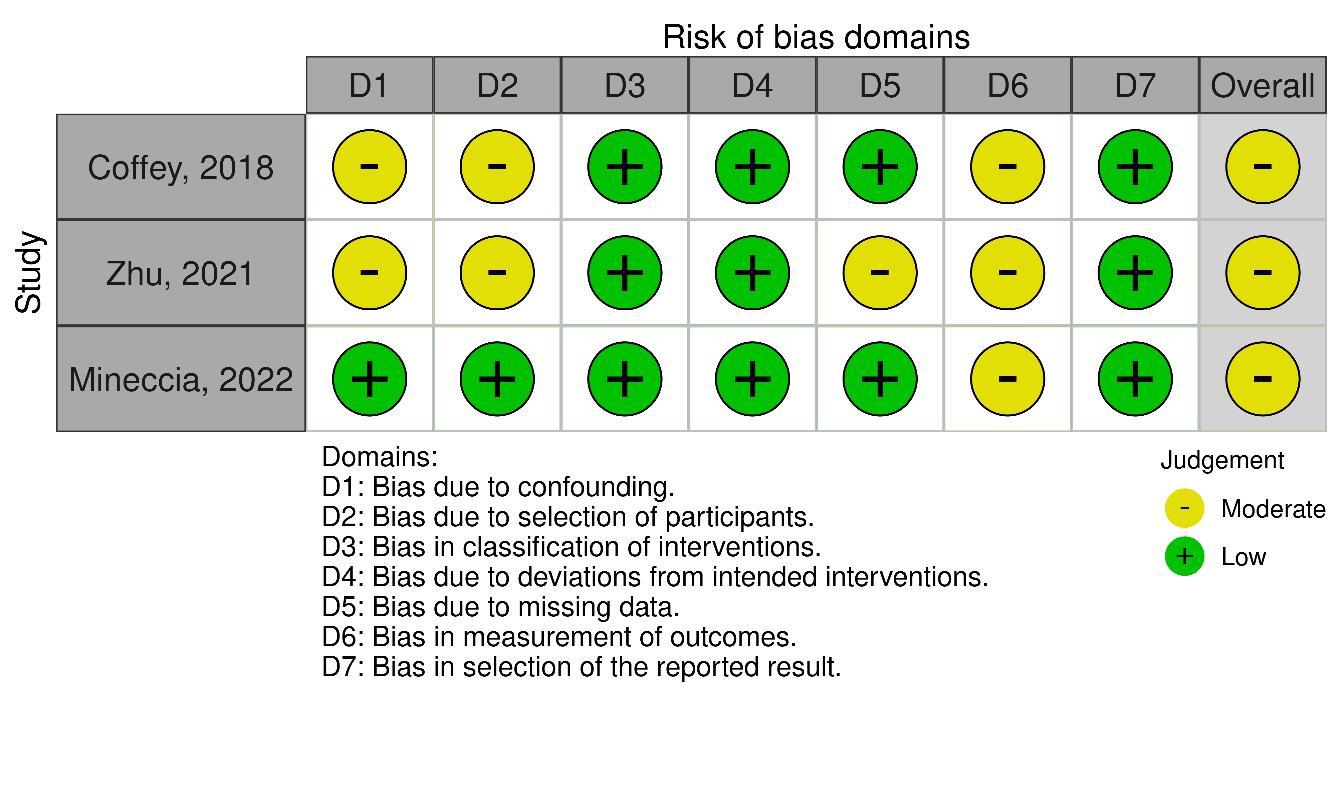


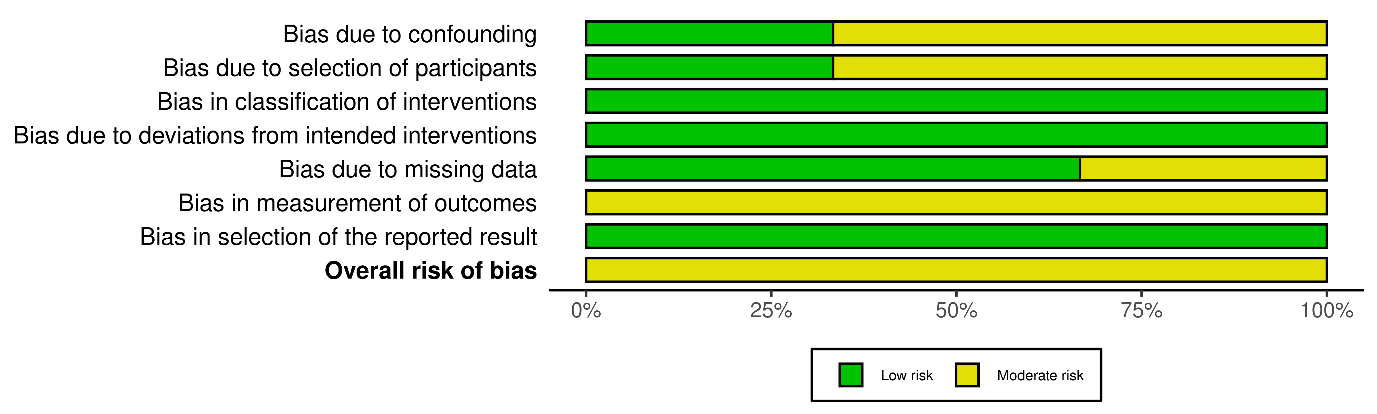


**Figure S2**. Risk of bias assessment (ROBINS–I tool) for the postoperative complications outcome


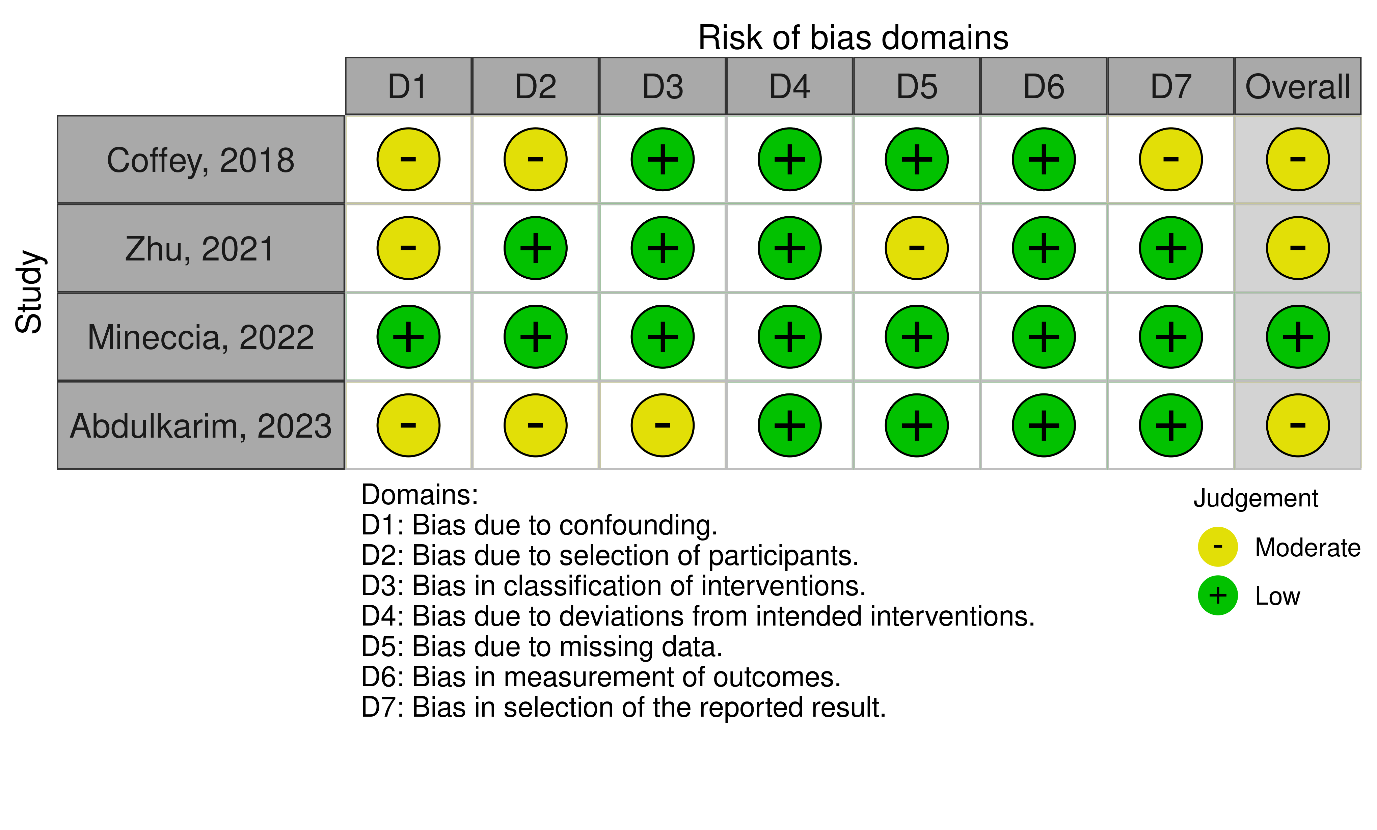


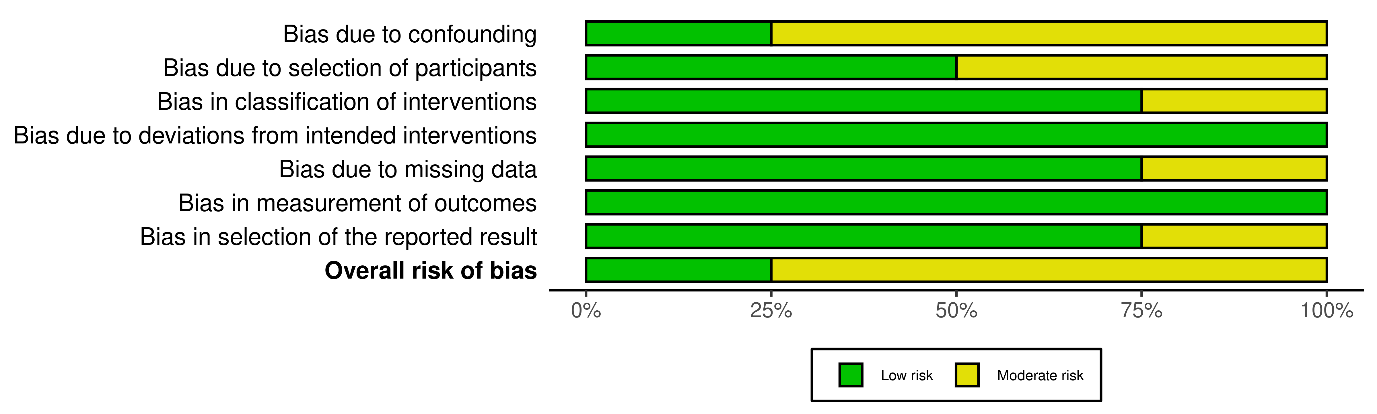


**Figure S3**. Risk of bias assessment (ROBINS–I tool) for the postoperative recurrence-free interval outcome


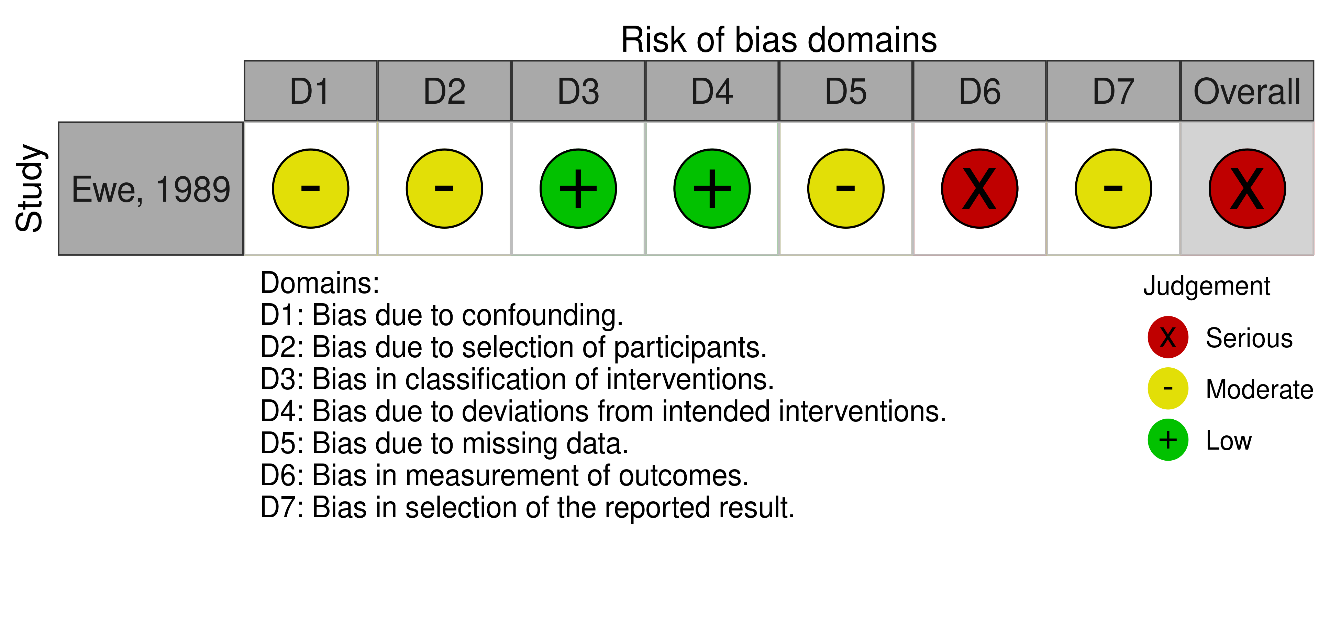

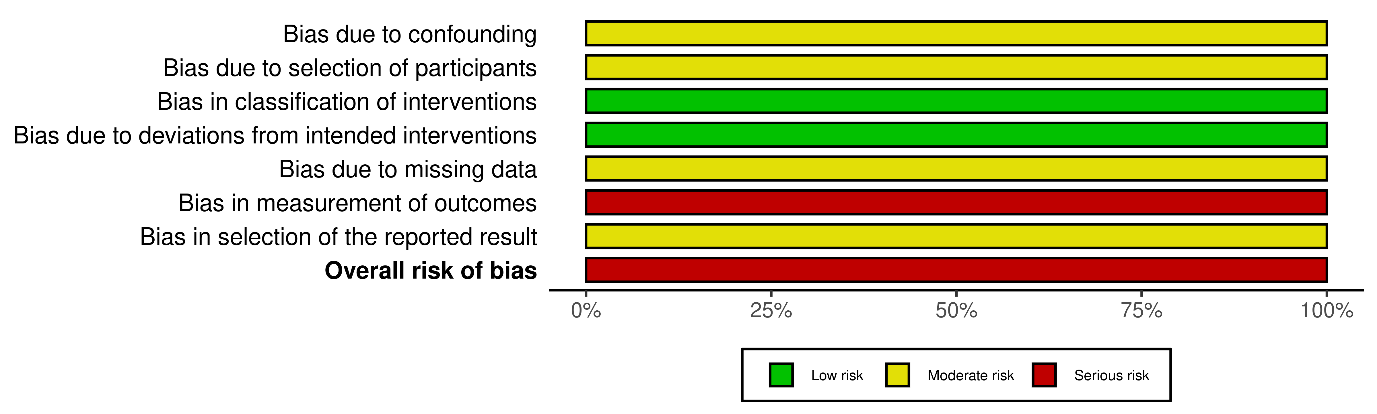


**Figure S4**. Risk of bias assessment (ROBINS–I tool) for the endoscopic recurrence outcome


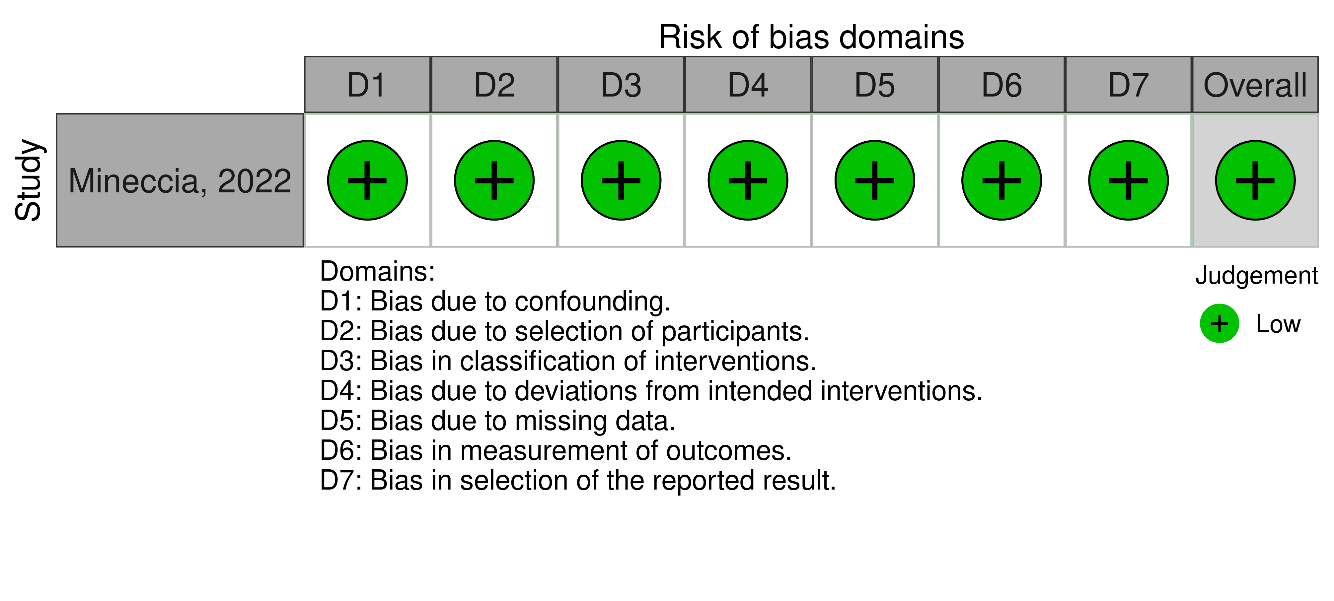


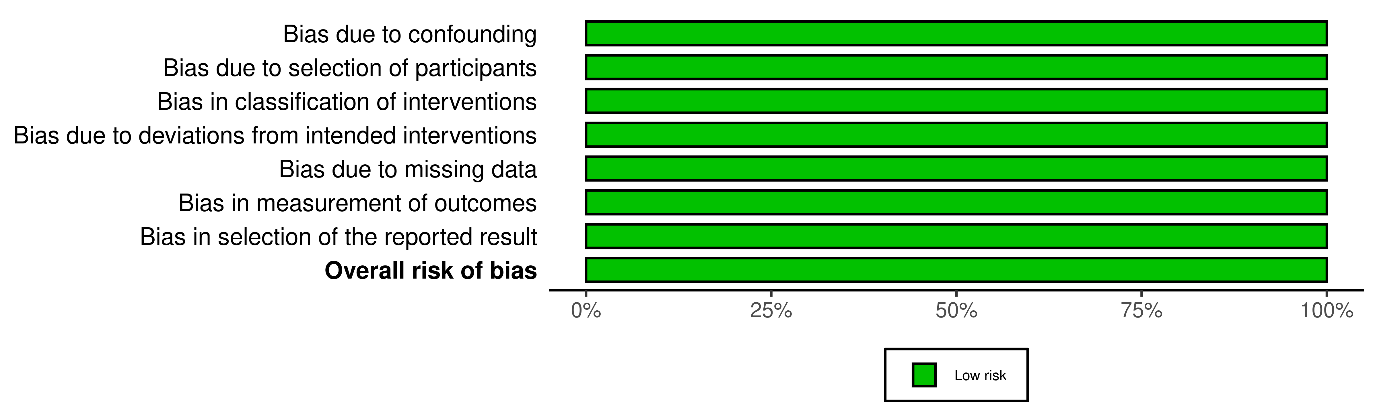


**Figure S5**. Risk of bias assessment (RoB2 tool) for the endoscopic recurrence outcome
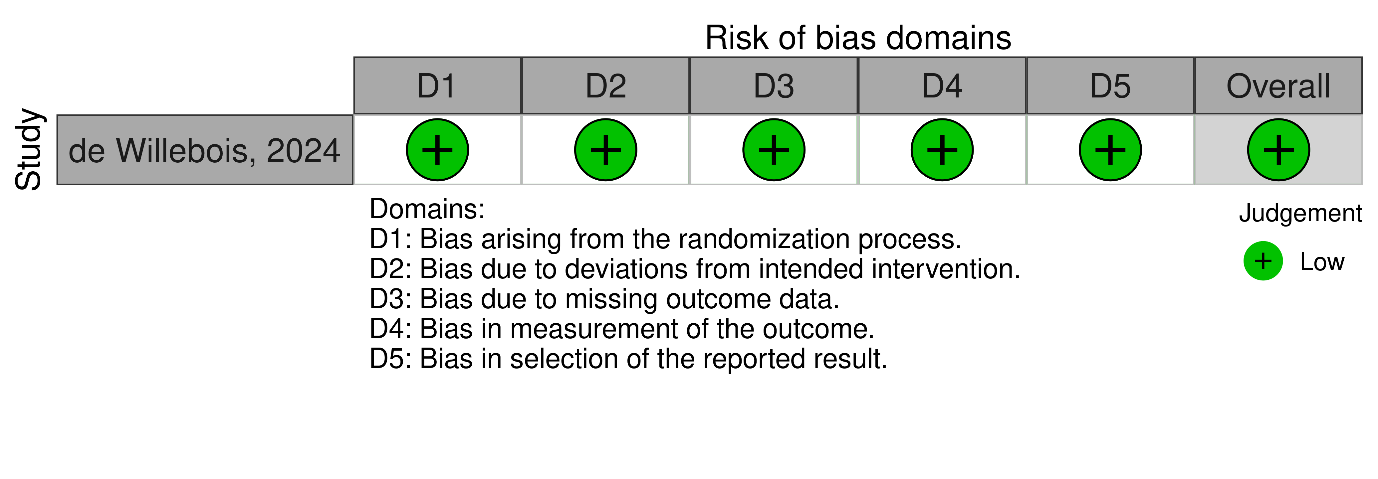


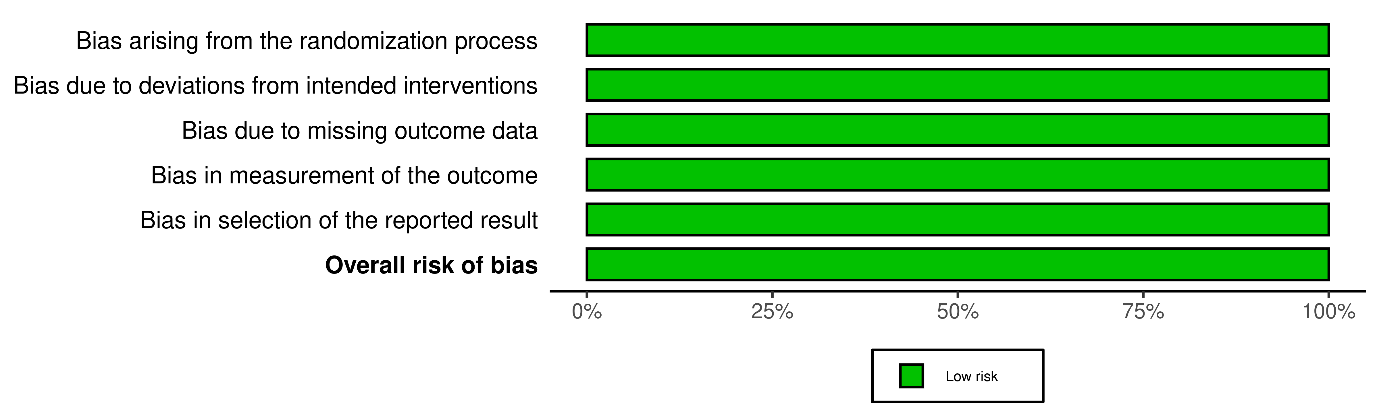


**Figure S6**. Risk of bias assessment (ROBINS–I tool) for the length of hospital stay outcome


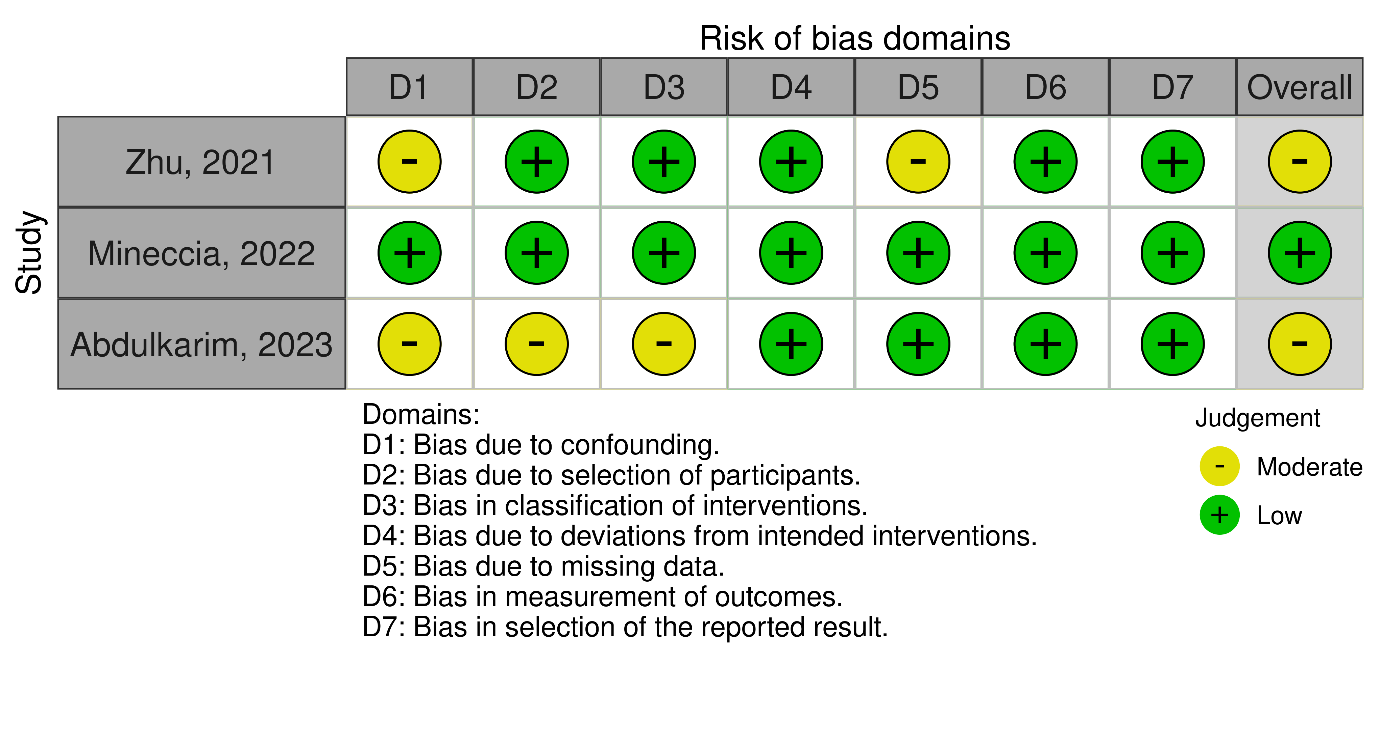


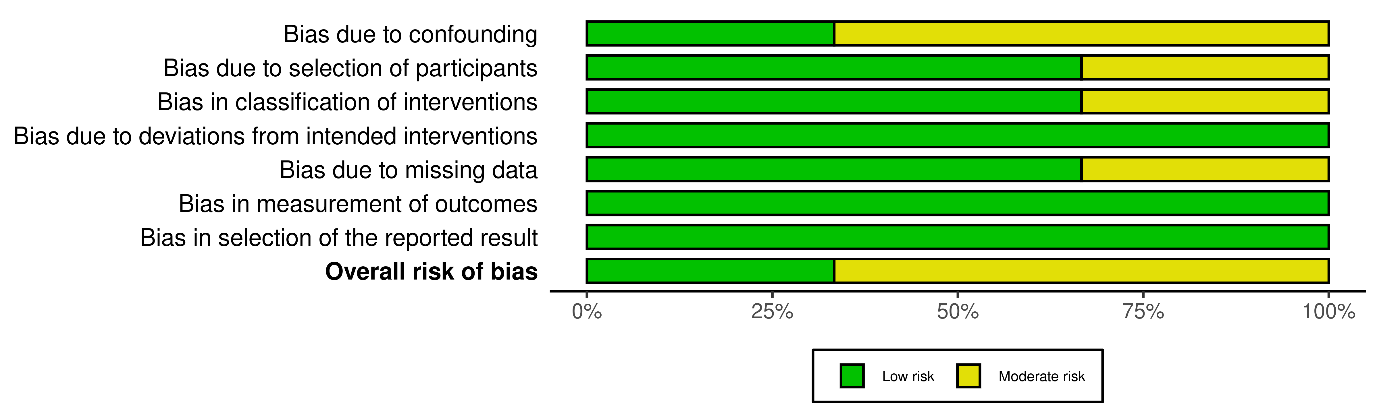


**Figure S7**. Risk of bias assessment (RoB2 tool) for the length of hospital stay outcome
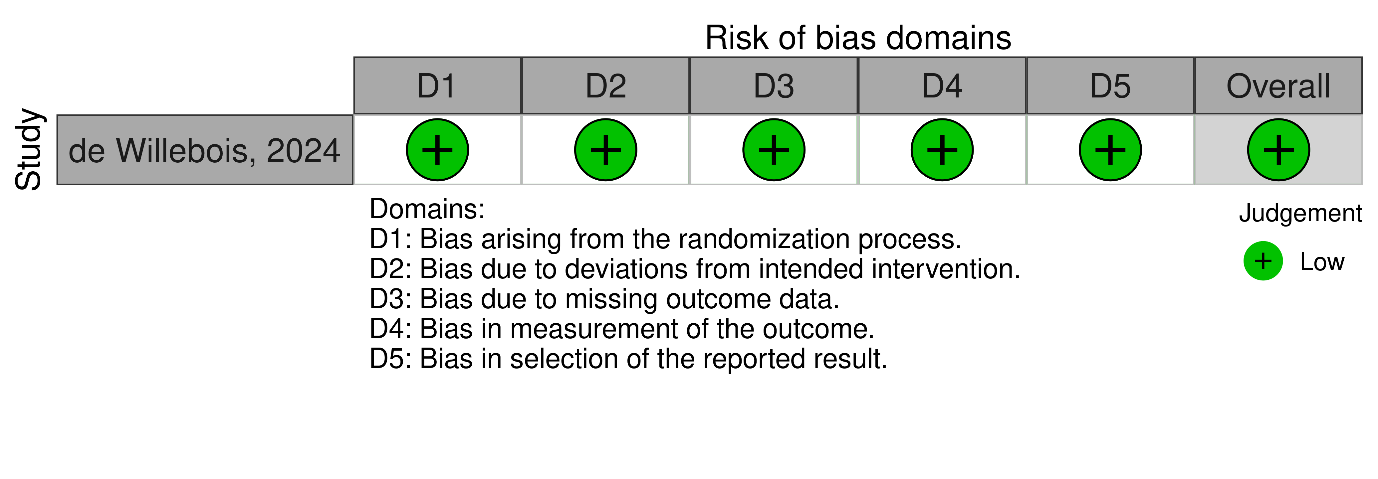


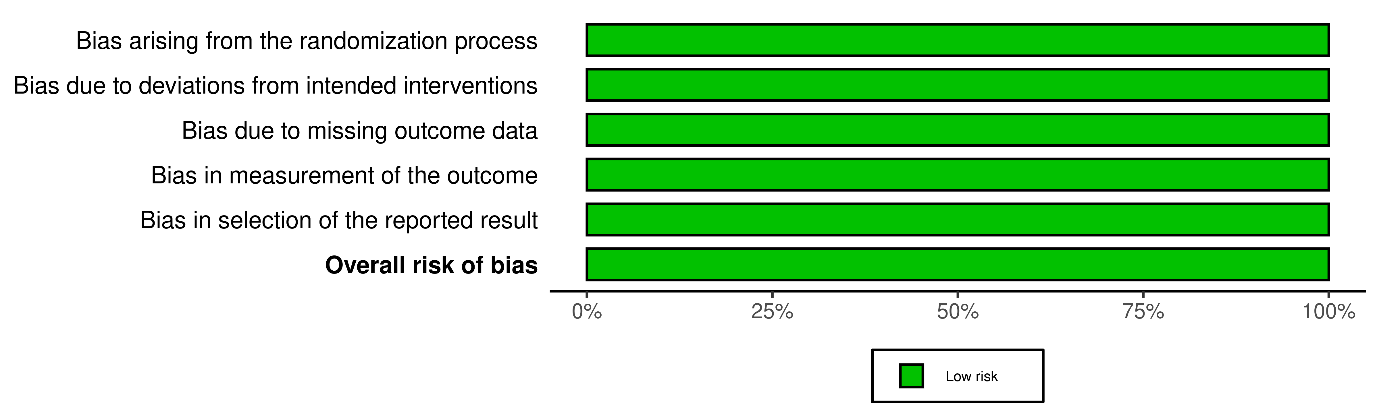


**Figure S8**. Risk of bias assessment (ROBINS–I tool) for the surgical site infection outcome


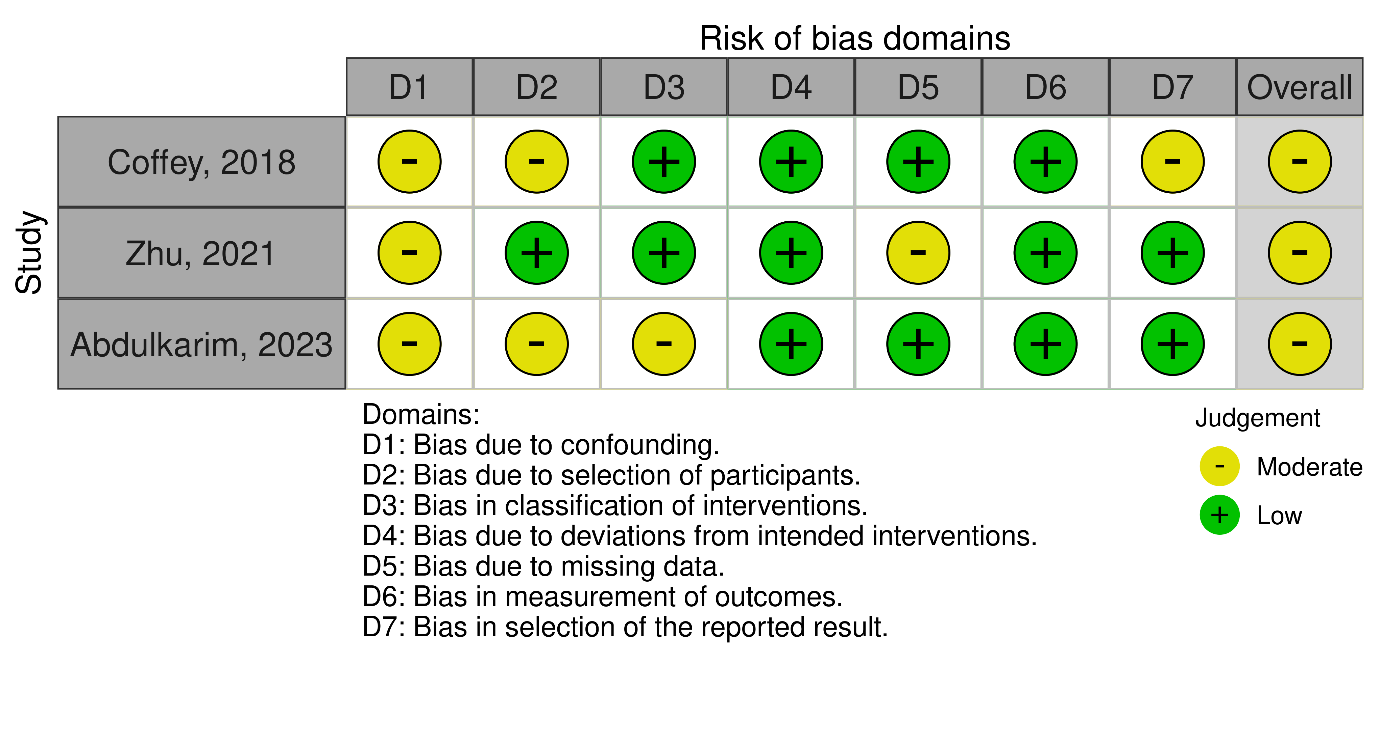


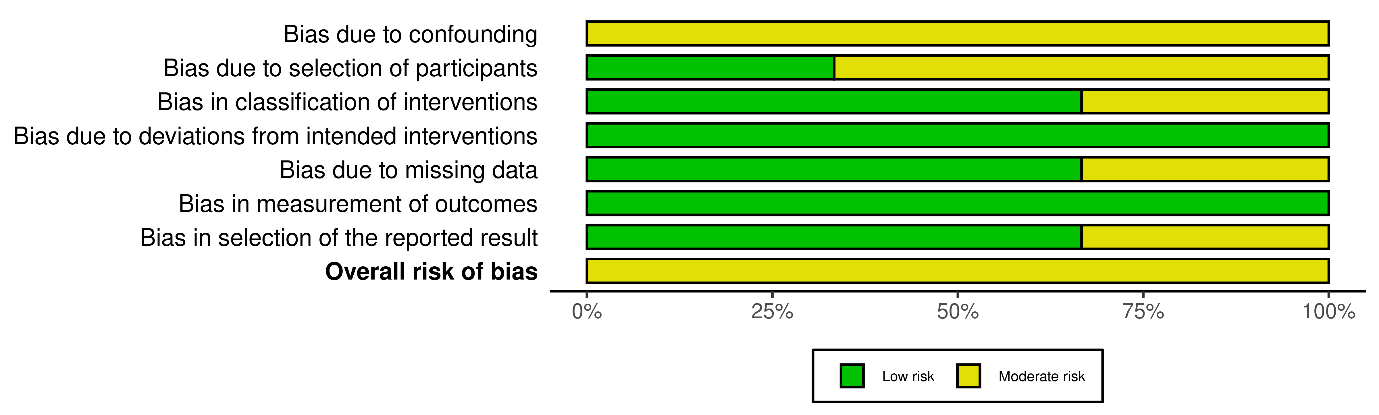


**Figure S9**. Risk of bias assessment (ROBINS–I tool) for the anastomotic leak outcome


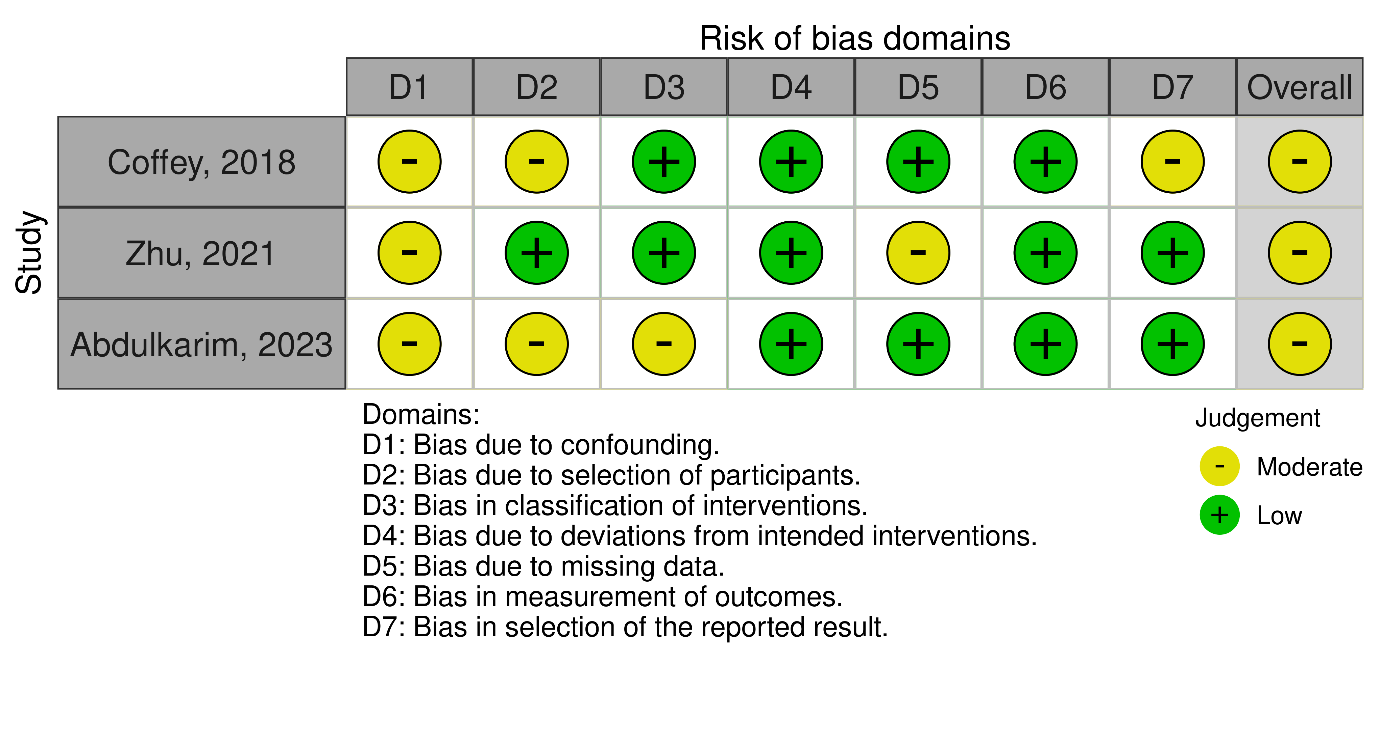


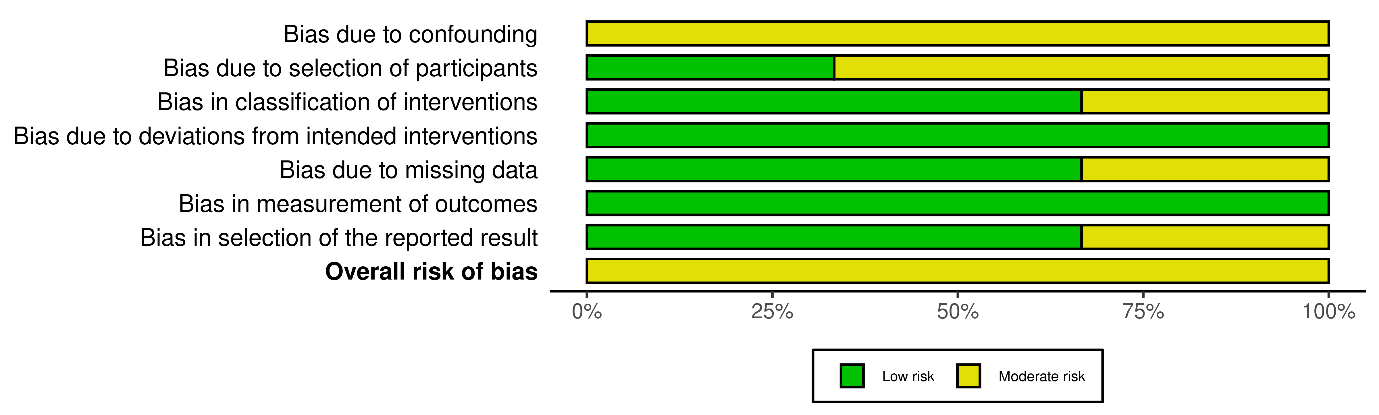


**Figure S10**. Risk of bias assessment (RoB2 tool) for the anastomotic leak outcome


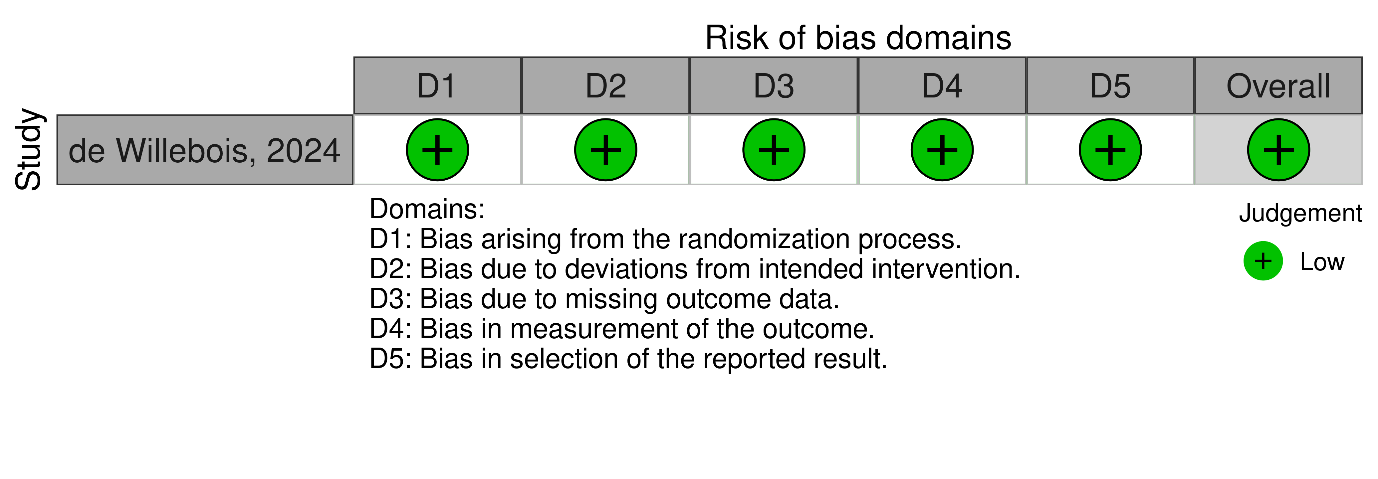


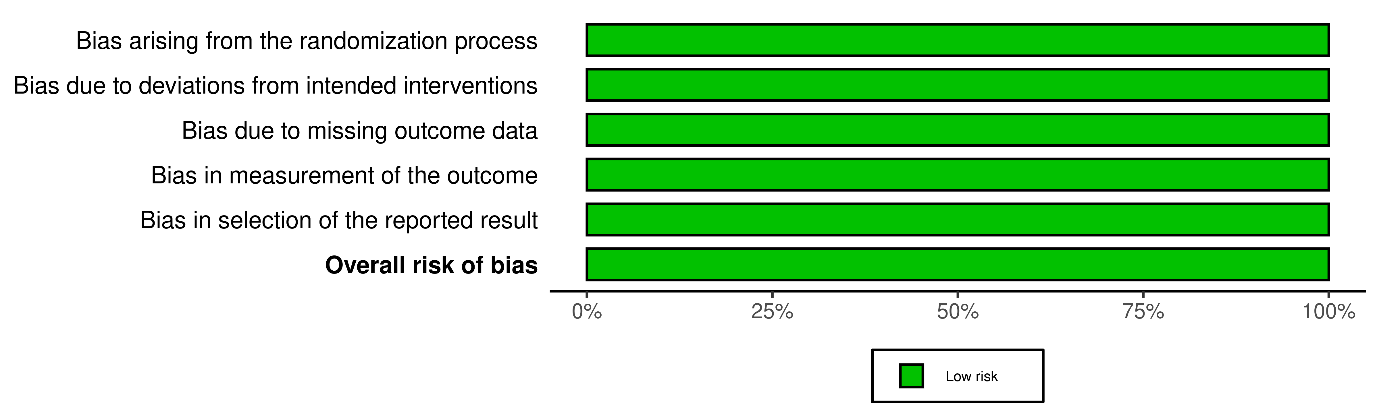


**Figure S11**. Risk of bias assessment (ROBINS–I tool) for the reoperation rate outcome


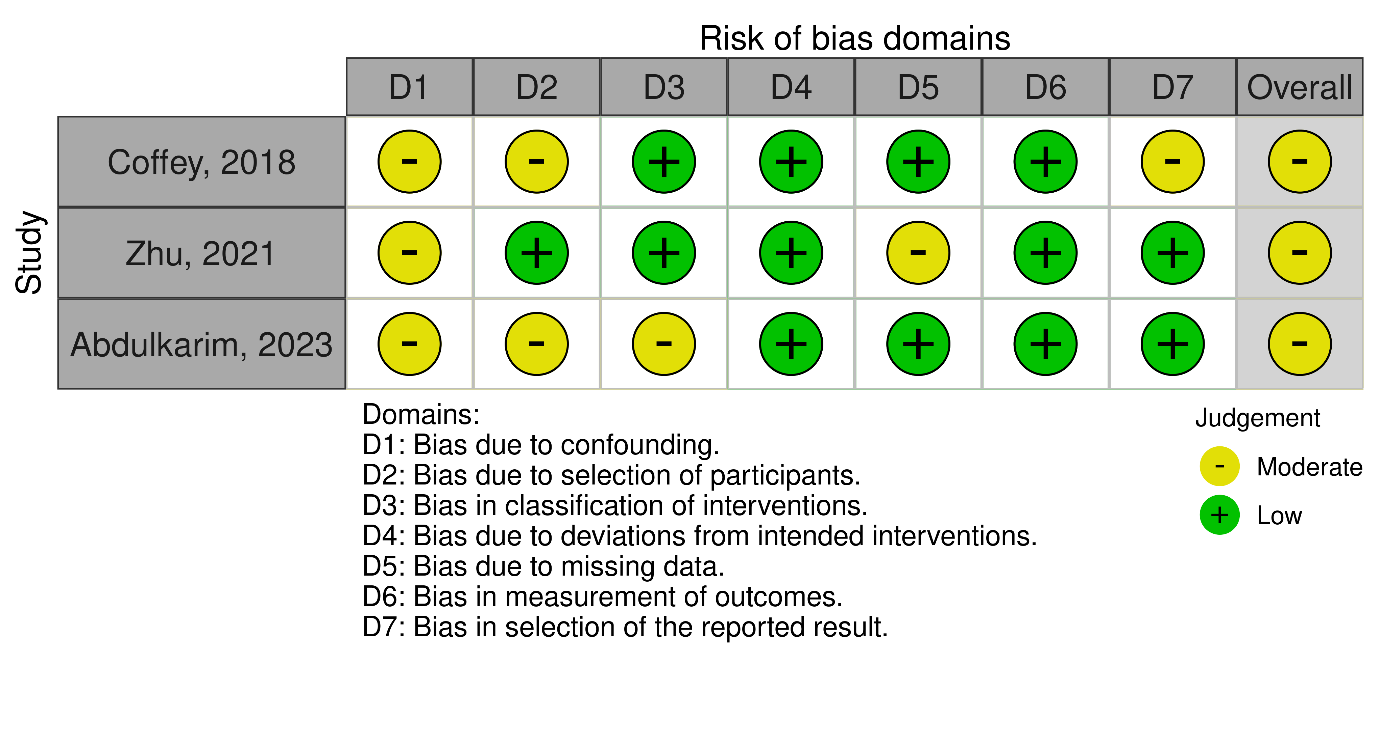


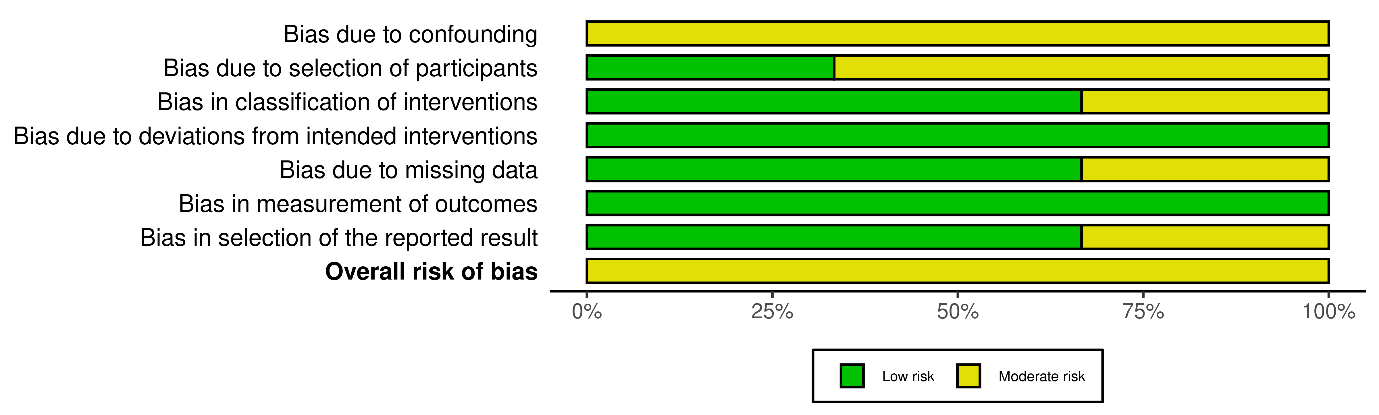


**Figure S12**. Risk of bias assessment (ROBINS–I tool) for operative time outcome


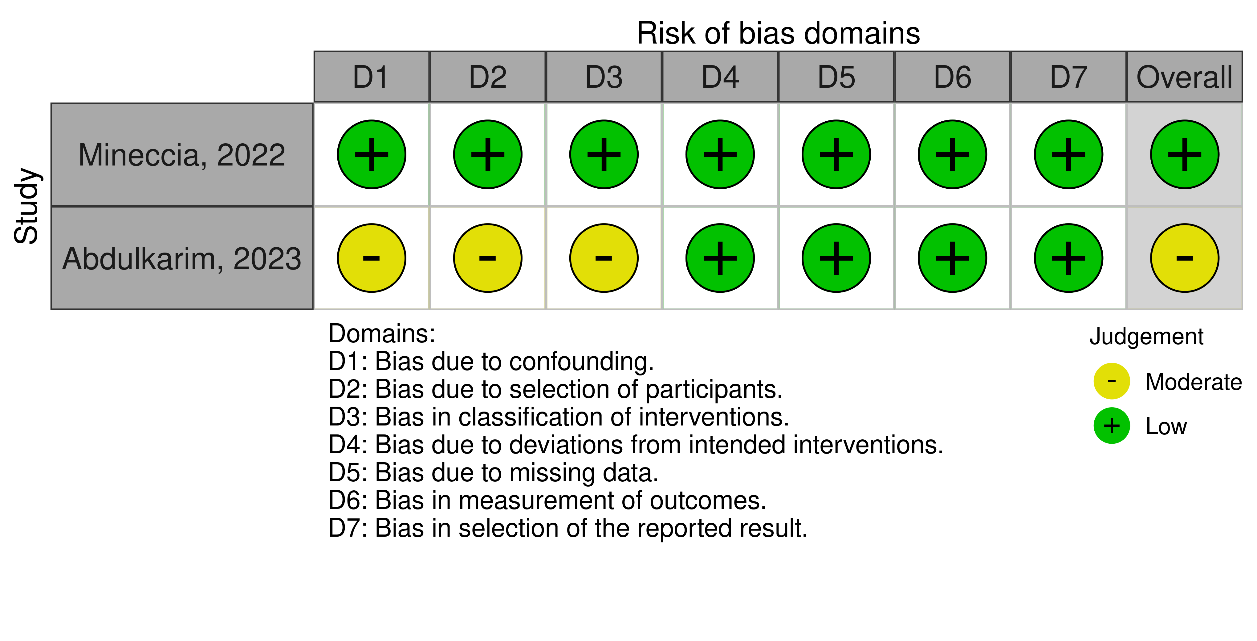


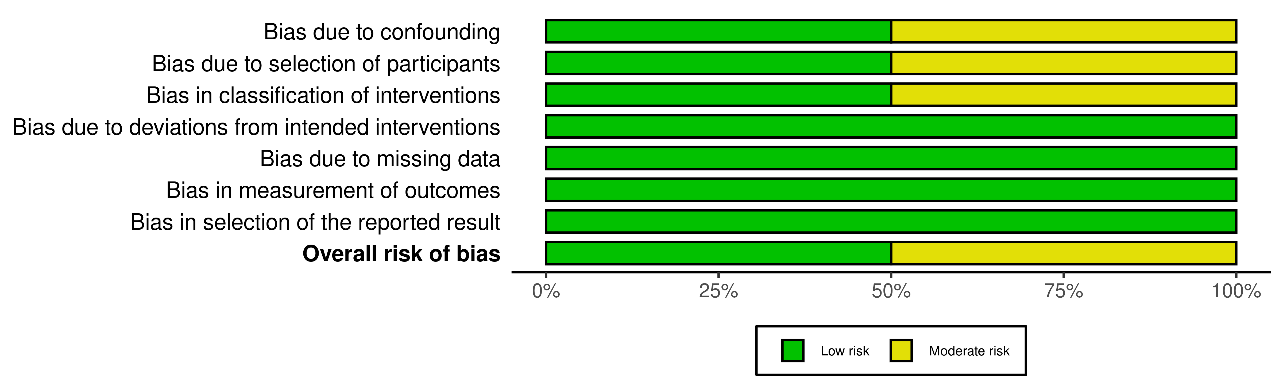


**Figure S13**. Risk of bias assessment (RoB2 tool) for the operative time outcome


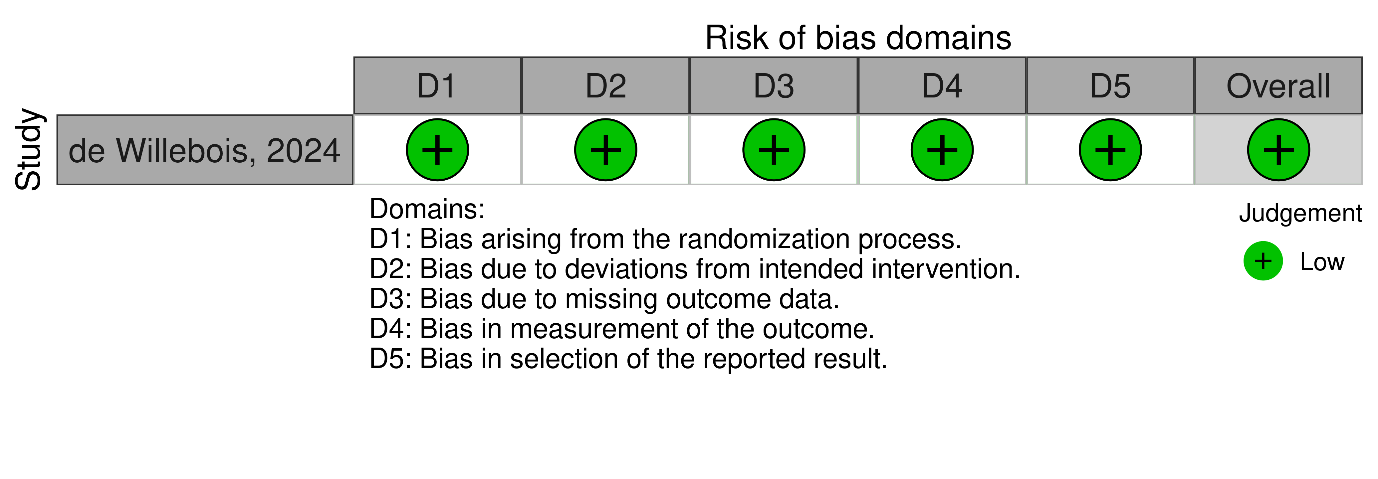


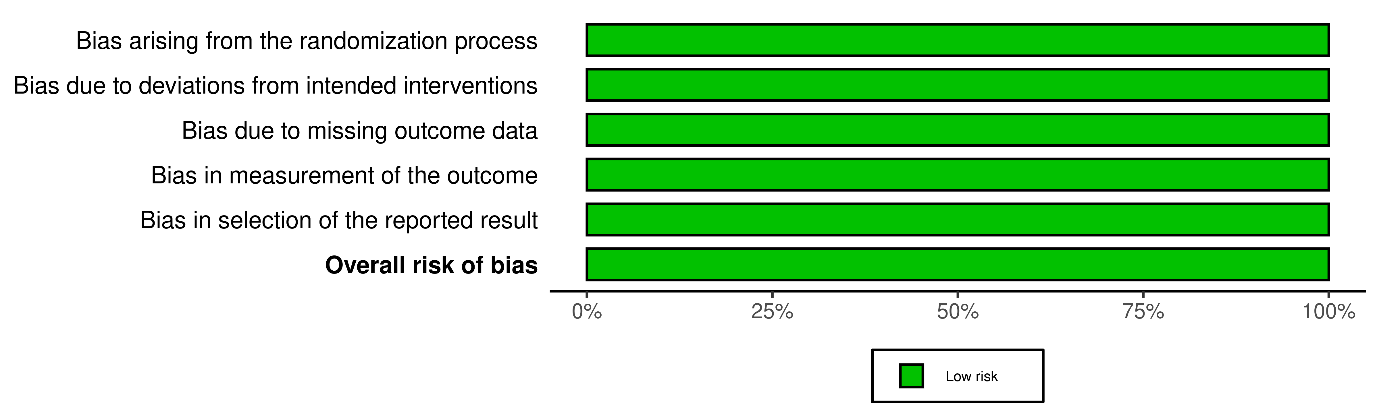


**Figure S14**. Risk of bias assessment (ROBINS–I tool) for the ultrasonographic recurrence outcome


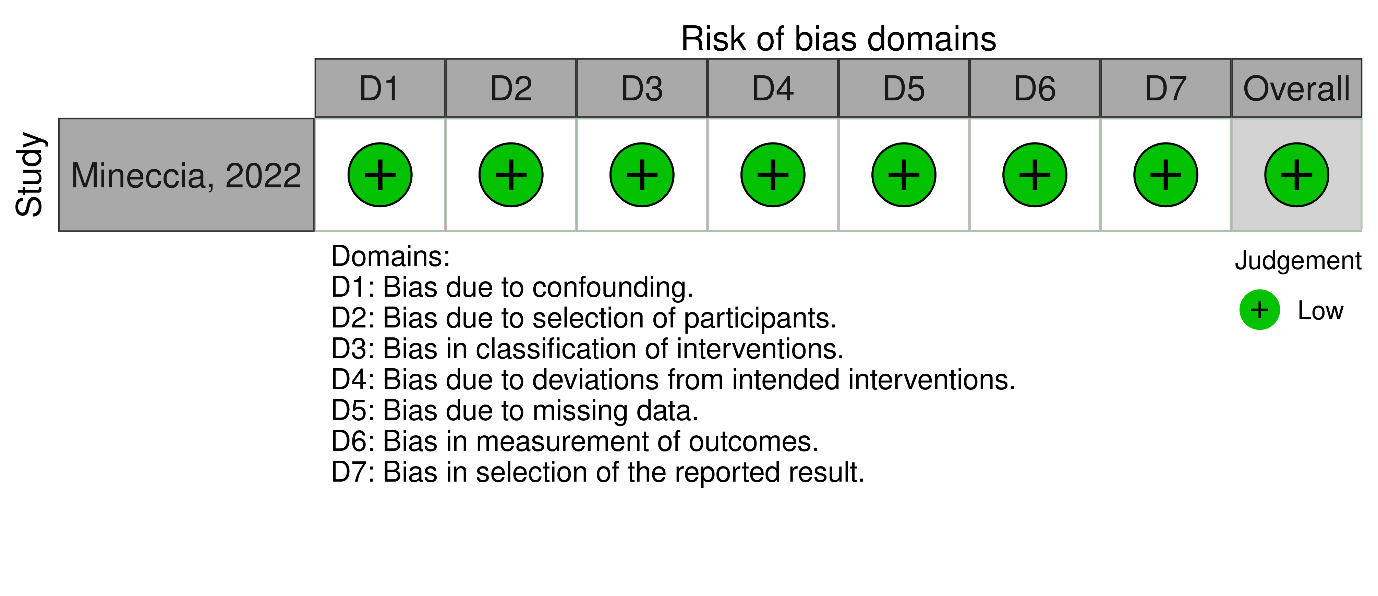


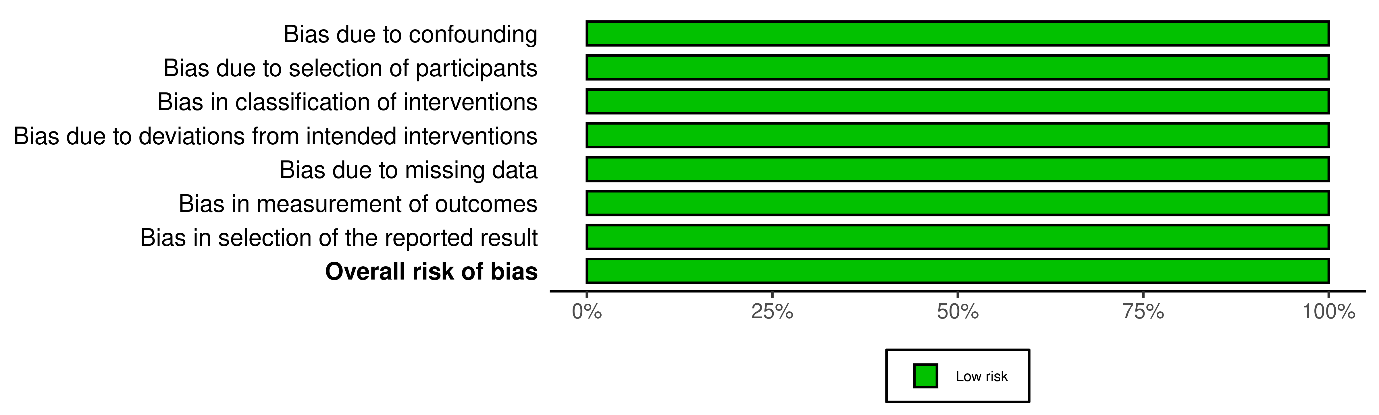


**Table S3.** Summary of findings table

| Certainty assessment | | | | | | | № of patients | | Effect | | Certainty | | Importance |  |
| --- | --- | --- | --- | --- | --- | --- | --- | --- | --- | --- | --- | --- | --- | --- |
| № of studies | **Study design** | **Risk of bias** | **Inconsistency** | **Indirectness** | **Imprecision** | **Other considerations** | **Extended mesenteric excision** | **limited mesenteric excision** | **Relative (95% CI)** | **Absolute (95% CI)** | |  |  | |
| Postoperative surgical recurrence | | | | | | | | | | | | | |  |
| 3 | non-randomised studies | serious^a^ | serious^b^ | not serious | very serious^c^ | strong association | 14/304 (4.6%) | 32/212 (15.1%) | **OR 0.30** (0.02 to 3.73) | **100 fewer per 1,000** (from 147 fewer to 248 more) | | ⨁◯◯◯ Very low^a,b,c^ | CRITICAL | |
| Overall postoperative complications | | | | | | | | | | | | | |  |
| 3 | non-randomised studies | serious^a^ | serious^d^ | not serious | serious^e^ | none | 80/304 (26.3%) | 68/212 (32.1%) | **OR 0.78** (0.33 to 1.82) | **52 fewer per 1,000** (from 186 fewer to 141 more) | | ⨁◯◯◯ Very low^a,d,e^ | IMPORTANT | |
| Surgical site infection | | | | | | | | | | | | | |  |
| 3 | non-randomised studies | serious^a^ | serious^,b^ | not serious | serious^c^ | none | 85/722 (11.8%) | 377/3177 (11.9%) | **OR 0.84** (0.30 to 2.36) | **17 fewer per 1,000** (from 80 fewer to 122 more) | | ⨁◯◯◯ Very low^a,b,c^ | IMPORTANT | |
| Anastomotic leak | | | | | | | | | | | | | |  |
| 3 | non-randomised studies | serious^a^ | serious^b^ | not serious | serious^c^ | none | 29/788 (3.7%) | 127/3242 (3.9%) | **OR 0.76** (0.09 to 785.00) | **9 fewer per 1,000** (from 36 fewer to 931 more) | | ⨁◯◯◯ Very low^a,b,c^ | IMPORTANT | |
| Anastomotic leak | | | | | | | | | | | | | |  |
| 1 | randomised trials | not serious | serious^b^ | not serious | serious^c^ | none | 5/66 (7.6%) | 1/65 (1.5%) | **OR 5.25** (0.60 to 46.20) | **60 more per 1,000** (from 6 fewer to 404 more) | | ⨁⨁◯◯ Low^b,c^ | IMPORTANT | |
| Reoperation | | | | | | | | | | | | | |  |
| 3 | non-randomised studies | serious^a^ | serious^b^ | not serious | serious^c^ | none | 31/622 (5.0%) | 131/3177 (4.1%) | **OR 1.09** (0.33 to 3.58) | **4 more per 1,000** (from 27 fewer to 92 more) | | ⨁◯◯◯ Very low^a,b,c^ | IMPORTANT | |
| Length of hospital stay | | | | | | | | | | | | | |  |
| 3 | non-randomised studies | not serious^a^ | serious^b^ | not serious | serious^c^ | none | 892 | 3269 | - | MD **0.33 lower** (1.8 lower to 1.15 higher) | | ⨁◯◯◯ Very low^a,b,c^ | IMPORTANT | |
| Length of hospital stay | | | | | | | | | | | | | |  |
| 1 | randomised trials | not serious | serious^b^ | not serious | serious^c^ | none | 66 | 65 | - | MD **0.35 higher** (0.56 lower to 1.27 higher) | | ⨁⨁◯◯ Low^b,c^ | IMPORTANT | |
| Operative time | | | | | | | | | | | | | |  |
| 3 | non-randomised studies | serious^a^ | serious^b^ | not serious | serious^c^ | none | 892 | 3274 | - | MD **0.98 lower** (17.96 lower to 16 higher) | | ⨁◯◯◯ Very low^a,b,c^ | IMPORTANT | |

**Abbreviations: CI:** confidence interval; **MD:** mean difference; **OR:** odds ratio;

#### Explanations

a. risk of bias - moderate level of bias for multiple criteria (ROBINS-I evaluation)

b. incosistency - serious (moderate level of heterogenity, although p>0.05, the CI are partially overlapping, the studies reported the same effect, but with large variation)

c. imprecision - low number of events, large CI, the CI also includes the value 1

d. inconsistency - although low level of heterogeneity, CI partially overlapping, large variation of effect size

e. imprecision - low number of events, large CI, includes value 1, but the effects is not crossing 0.75
